# Multi-Lateral Teleoperation Based on Multi-Agent Framework: Application to Simultaneous Training and Therapy in Telerehabilitation

**DOI:** 10.3389/frobt.2020.538347

**Published:** 2020-11-11

**Authors:** Iman Sharifi, Heidar Ali Talebi, Rajni R. Patel, Mahdi Tavakoli

**Affiliations:** ^1^Electrical Engineering Department, Amirkabir University of Technology, Tehran, Iran; ^2^Electrical & Computer Engineering Department, Western University, London, ON, Canada; ^3^Electrical & Computer Engineering Department, University of Alberta, Edmonton, AB, Canada

**Keywords:** tele-rehabilitation system, robotics, non-linear control, multi-agent systems (MAS), force control, cooperative teleoperation

## Abstract

In this paper, a new scheme for multi-lateral remote rehabilitation is proposed. There exist one therapist, one patient, and several trainees, who are participating in the process of telerehabilitation (TR) in this scheme. This kind of strategy helps the therapist to facilitate the neurorehabilitation remotely. Thus, the patients can stay in their homes, resulting in safer and less expensive costs. Meanwhile, several trainees in medical education centers can be trained by participating partially in the rehabilitation process. The trainees participate in a “hands-on” manner; so, they feel like they are rehabilitating the patient directly. For implementing such a scheme, a novel theoretical method is proposed using the power of multi-agent systems (MAS) theory into the multi-lateral teleoperation, based on the self-intelligence in the MAS. In the previous related works, changing the number of participants in the multi-lateral teleoperation tasks required redesigning the controllers; while, in this paper using both of the decentralized control and the self-intelligence of the MAS, avoids the need for redesigning the controller in the proposed structure. Moreover, in this research, uncertainties in the operators' dynamics, as well as time-varying delays in the communication channels, are taken into account. It is shown that the proposed structure has two tuning matrices (*L* and *D*) that can be used for different scenarios of multi-lateral teleoperation. By choosing proper tuning matrices, many related works about the multi-lateral teleoperation/telerehabilitation process can be implemented. In the final section of the paper, several scenarios were introduced to achieve “Simultaneous Training and Therapy” in TR and are implemented with the proposed structure. The results confirmed the stability and performance of the proposed framework.

## 1. Introduction

Telerehabilitation (TR) can be regarded as a telemedicine branch. While this field is considerably new, it is used in developed countries and has expanded rapidly. Patients living in remote areas where conventional rehabilitation services may not be readily available, will benefit from this technology. TR technologies are open to the patient with existing devices, such as laptops or mobile phones. In such methods, video calls, web-based and mobile apps can be used as well (Bostrom et al., 2020). TR typically lowers the costs of both healthcare services and patients compared to conventional inpatient or individual-to-person rehabilitation. Few studies have been conducted on the economic aspects of TR in which the cost of hospitalization in clinics is significantly reduced (Peretti et al., 2017; Schröder et al., 2019). TR is mainly applied to the physiotherapy process, and neural rehabilitation is used to monitor the rehabilitation process of stroke patients (Gal et al., 2015; Mani et al., 2017). The TR process is also performed with neuro-rehabilitative techniques, such as telemonitoring of cardiovascular parameters including oxygen saturation, ECG, and blood pressure for patients with heart disease (Tousignant et al., 2019). These techniques belong to another branch of telemedicine called telemonitoring, which has significantly expanded in recent years (Batalik et al., 2020). TR for regular training sessions can be accomplished several times in the week as oppose to clinical rehabilitation, which is usually done once or twice a week. TR can also be done individually or in groups (Rogante et al., 2015). These groups include a large number of patient, trainees, and therapists (Sharifi et al., 2017). Interactive tools, such as gamification can increase motivation while the training/therapy process is in progress. Also, TR, if done at home, can support more frequent exercises both in terms of numbers in the week and duration length (Peretti et al., 2017). Furthermore, TR can be delivered with haptic-enabled robotic manipulators in which the patient can interact directly with them. Therefore, the TR process can be performed in virtual reality, while the rehabilitation for neurological conditions is done using robots and gamification (Larson et al., 2014). Also, due to the presence of position and force sensors in the haptic-enabled devices, the progress of a patient's treatment can be shown numerically and on a graph (Schröder et al., 2019).

The specific idea of the proposed TR methods in this paper, came to the minds of the authors after frequent presence in physiotherapy clinics, observing the rehabilitation process, observing the training of trainees, and consulting with physiotherapists. For the implementation of the idea, the project was divided into three phases. In the first phase, the controller should be designed to involve several robots in the rehabilitation process, and to study its feasibility on non-homogeneous and conventional manipulators for the teleoperation process. In the second phase, dedicated manipulators will be built for rehabilitation operations, and the results of the first phase will be studied on it. In the third phase, the products of the previous phases will be tested in the clinic and on real patients. This article will cover the first phase of our TR project, and the rest of the phases will be reported in separate articles. So, in this paper, the concept of collaborative teleoperation and its usage in TR will be extended. All the participants in the experiments of this article are students and non-patients. In the continuation of this introduction, the available researches in the teleoperation and, the advances in robotic rehabilitation that have been made in this field, will be discussed.

Recently, teleoperation frameworks have incredibly extended human control capacities in critical or dangerous situations (Ferre et al., 2007). Up until this point, many propelled control schemes have been accounted for teleoperation frameworks (e.g., Nuño et al., 2009, 2011; Chan et al., 2016; Jafari and Spong, 2017) to give some examples, where a large portion of the previously mentioned examinations concern the control of single-master, single-slave setups. Given that numerous viable assignments cannot be finished by only a single robot. For example, conveying a heavy or delicate thing needs more than one manipulators to do more precise tasks. Another vital concern is the method by which to teleoperate various slave robots in a cooperative configuration. Presently, an ever-increasing number of researches have been committed to this field (Mohajerpoor et al., 2013; Zhai and Xia, 2016), which for the most part, incorporates single-master multi-slave and multi-master multi-slave arrangements (Khademian and Hashtrudi-Zaad, 2013; Zhai and Xia, 2016). Moreover, the multilateral cooperative teleoperation framework has quickly risen in numerous conceivable applications that range from industrial assembly tasks to material handling in perilous situations and afterward to TR tasks for neurological lesions.

A stroke and spinal cord injuries are two principal purposes behind neurological lesions. Since 2008, just in the US, adding up to the cost of stroke is 34.3 billion dollars, and in 2016 it was estimated to be 69.1 billion dollars (Writing et al., 2016). In the light of the results of experiments, frequent movement repetition challenges regular physiotherapeutic methods for the motor rehabilitation of the central paretic forearm in the way that early starting of dynamic developments has a superior result than decreasing spasticity in the recovery of patients (French et al., 2016). This means task-oriented repetitive movements have a direct positive effect on muscle strength enhancement and development in neurologically injured patients. Robotics and automation technology are capable of assisting and enhancing rehabilitation by acquiring a high number of moves in repetition (Atashzar et al., 2017).

The traditional physiotherapy has several limitations with respect to the manually-assisted therapy criteria. In traditional physiotherapy, it is complicated to teach a trainee. Also, evaluating the trainee's performance is laborious and time-consuming. Training consistency is tied to therapist experience and performance. Unlike conventional methods, the rehabilitation procedure can be automated by implementing robotic devices, which increases device training sessions and process duration. As mentioned earlier, robotics therapy can be a practical and highly motivational context for virtual reality applications, and therefore treatment can achieve better results (Nef et al., 2016).

There are typically two types of rehabilitation robots, the first is the robots mounted on the end-effector, and the second is the exoskeletons. Exoskeletons have a resemblance to human anatomy and could be actuated by specific methods, whereas robots with end-effectors could be in any configuration. There is some kind of upper-extremity rehabilitation of exoskeleton robots like MAHI Exo-II, ETS-MARS, and CADEN-7 and some form of end-effector like MIT-MANUS and MIME (Krebs et al., 1998; Pehlivan et al., 2012; Niyetkaliyev et al., 2017; Brahmi et al., 2019; McDonald et al., 2020).

A major problem in multi-lateral teleoperation systems occurs when the number of robots involved in the interactions is increased. In this situation, the control design and stability analysis problems may become more challenging. The self-intelligence that exist between multiple agents interacting with each other in a MAS can be a key to solve the mentioned problem.

A multi-agent system consists of agents who can interact with their neighbors while making decisions. The shared information between the agents will help them together achieve the desired objective. The goal could be synchronization, coverage, or consensus (Sharifi et al., 2016; Wang et al., 2017; Wen et al., 2017; Xiang et al., 2017). One of the fundamental goals in multi-agent systems is synchronization, which means an agreement between agents over a target given the network's limitations (Peng et al., 2014; Sun, 2016). Consequently, the concept of remote multi-lateral TR based on MAS synchronization was previously introduced in Sharifi et al. (2016). It has been shown that the issue of bilateral teleoperation can be viewed as a problem of synchronization, in which the MAS synchronizes the operators' forces and positions. Although, the similar concept was defined in Spong and Chopra (2007) and Abdessameud and Tayebi (2011), it was considered that the dynamics of manipulators are Lagrangian without the effects of exerted external force. However, in TR systems, the concept of external force (operator forces) is not ignorable.

Based on these facts, in this paper, a new control scheme based on MAS is developed for several rehabilitation scenarios, that can deal with non-linear uncertain manipulators. Moreover, the scheme has the ability to design a desired hand force for each operator, which helps deal with training and therapy, concurrently. This new methodology is called “simultaneous training and therapy.” Additionally, the concept of decentralized controllers is introduced for multi-lateral teleoperation systems. Through decentralized control, the reliability of the systems increases while the number of communication links decreases (Hou et al., 2009; Hernández-Méndez et al., 2016). Because of the self-intelligence feature in the MAS, the delay does not distribute between agents synergistically (Cao et al., 2013). Furthermore, time- varying delays in communication links are considered in the current work, which allows the implementation of a multi-lateral teleoperation system through the internet or other communication networks (Chopra et al., 2003; Wu et al., 2017). The structure of a dual-user teleoperation system with a shared environment is one of the most popular structure in multi-lateral teleoperation systems in recent years (Khademian and Hashtrudi-Zaad, 2012; Li et al., 2015; Shamaei et al., 2015; Hashemzadeh et al., 2016). The authority sharing structures in those papers can be regarded as a special case of the current research by applying matrices *D, L*, and *P* ≥ 0 that are investigated in section 6.

The remainder of the paper is organized as follows. Section 2, presents mathematical preliminaries concerning, the MAS, properties of serial link manipulators and multi-lateral teleoperation systems. Moreover, it introduces correspondence between the MAS, and multi-lateral teleoperation systems. Section 3 presents a new centralized controller for a multi-lateral teleoperation system. Throughout section 5, the controller is strengthened with a passivity-based adaptive control scheme in the presence of uncertainty in both of the environment and the operator. Afterward, in section 5, the decentralized controller based on the intelligence of a multi-agent framework is introduced to solve the problem of time-varying in communication networks while minimizing the number of communication links. Section 6 shows the relevance of the proposed method and the similar existing methods for multi-lateral teleoperation/telerehabilitation, such as “teach and repeat” and “assist as needed” (Staubli et al., 2009; Babaiasl et al., 2016; Luo et al., 2019). Moreover, it proposes novel schemes for multi-lateral remote rehabilitation systems and experimentally investigates them. Finally, section 7 discusses the conclusions and future works.

## 2. Mathematical Preliminaries

A brief introduction about the terms and expressions used in the proposed structure is presented in this section. The first subsection relates to MAS, and the second subsection explores the serial link manipulators. Afterward, the third subsection presents the terms and equations for multi-lateral teleoperation systems. Lastly, in the fourth subsection, the multi-lateral teleoperation approach based on the MAS is implemented.

### 2.1. MAS Framework

The theory of graphs is a powerful tool to study MAS and its behaviors. An undirected G graph on the vertex set V=1,2,…,N contains V and a set of unordered pairs E={(i,j):i,j∈V} which are called the edges of G. Two vertices are called adjacent, if there is a line between them.

Consider a system consisting of *N* agents. The position of the *i*th agent is denoted by *x*_*i*_ for *i* = 1, …, *N*. Considering the *N* agents as the vertices in V, the relationships between the *N* agents can be explained by a simple and undirected graph G.

The weighted adjacency matrix $A=\left[\alpha_{ij}\right]\in {\text{R}}^{n\times n}$ for the graph G is denoted such that α_*ij*_ = 0 if there exists no input from the *j*th agent to *i*th agent; otherwise, α_*ij*_ ≠ 0.

The degree matrix $D=\text{diag}\left\{d_{1},d_{2},\ldots ,d_{N}\right\}\in {\text{R}}^{N\times N}$ is a diagonal matrix, where diagonal elements are $d_{i}=\sum_{j=1}^{N}\alpha_{ij}$ for *i* = 1, …, *N*. Then, the weighted graph's *Laplacian* matrix is defined as L≜D-A. If there is a path between any two vertices, a directed graph is connected.

Remark 1. *The Laplacian L has real eigenvalues for graph* G, *which can be ordered in succession as* 0 = λ_1_(*L*) ≤ λ_2_(*L*) ≤ … ≤ λ_*n*_(*L*) ≤ 2*d*_*max*_. *The smallest eigenvalue is always zero, and the second smallest eigenvalue* λ_2_(*L*) *is called the graph's algebraic connectivity (Olfati-Saber et al., 2007)*.

Remark 2. *If there exists a MAS with a connected graph and positive weights, then a vector γ (with positive elements) exists such that it satisfies γ*^*T*^*L* = 0, *where the vector γ is defined as* $\gamma ={\left[\gamma_{1},\ldots ,\gamma_{N}\right]}^{T},\;\gamma_{i}>0,\;i=1,\;\ldots ,\;N$ *for the N agents scenario (Cao et al., 2013; Zhou et al., 2014)*.

The latter remark points to a fundamental matter, which is the existence of a connected graph. This principle is instrumental in our proofs of stability as well as experimentations in section 6, for the Laplacian matrix (*L*).

### 2.2. Serial Link Manipulator Properties

Some properties of serial link manipulators, which can be found in Sciavicco and Siciliano (2012) are written in this subsection. The robot that interacts with the slave(s) and master(s) in teleoperation systems is regarded as *n*-DOF serial links with totally revolute joints. The related non-linear dynamics of these robots can be defined as follows.

(1) $$\begin{array}{l} M_{i}\left(q_{i}\right){\ddot{q}}_{i}+C_{i}\left(q_{i},{\dot{q}}_{i}\right){\dot{q}}_{i}+g_{i}\left(q_{i}\right)=-\tau_{{ext}_{i}}+\tau_{c_{i}} \end{array}$$

in which $M_{i}\left(q_{i}\left(t\right)\right)\in {\text{R}}^{n\times n},$ $C_{i}\left(q_{i}\left(t\right),{\dot{q}}_{i}\left(t\right)\right)\in {\text{R}}^{n\times n},$ and $g\left(q_{i}\left(t\right)\right)\in {\text{R}}^{n\times 1}$ are inertia matrix, Coriolis/centrifugal matrix, and gravitational vector, respectively. In addition, $q_{i},{\dot{q}}_{i}$, and ${\ddot{q}}_{i}\in {\text{R}}^{n\times 1}$ for *i* = 1, 2, …, *N* are the joint angle, angular velocities, and angular accelerations of the *i*th robot (Sharifi et al., 2011).

If the *i*th robot is interacting directly with the human, then τ_*ext*_*i*__ = −τ_*h*_*i*__ (torque applied by the operator of *i*th robot). If the one is interacting with the environment, then τ_*ext*_*i*__ = τ_*e*_*i*__ (torque applied by the *i*th environment). Finally, $\tau_{c_{i}}\in {\text{R}}^{n\times 1}$ are control torques for the master and slave robots.

Property 1. *For manipulators with totally revolute joints, the Coriolis/centrifugal terms are bounded, and the form of the bounds are as follows*

$${\left\|C_{i}\left(q_{i},x\right)y\right\|}_{2}\leq {\left\|x\right\|}_{2}{\left\|y\right\|}_{2}$$

*The fact can easily be generalized to the augmented equation that diagonally puts the C*_*i*_(*q*_*i*_, *x*)*y matrices for i* = 1, …, *N together, like the one in (4), that is*

$${\left\|C.Y\right\|}_{2}\leq {\left\|X\right\|}_{2}{\left\|Y\right\|}_{2}$$

*in which*, $X={\left[x_{1}^{T},\ldots ,x_{N}^{T}\right]}^{T}$, $Y={\left[y_{1}^{T},\ldots ,y_{N}^{T}\right]}^{T}$, *and* C *is a diagonal matrix and is defined as* $C=\mathrm{diag}\left\{C_{1}\left(q_{1},x_{1}\right),C_{2}\left(q_{2},x_{2}\right),\ldots ,C_{N}\left(q_{N},x_{N}\right)\right\}$.

Property 2. *The relationship between the Coriolis/centrifugal and the inertia matrix for a serial manipulator is* ${Ṁ}_{i}\left(q_{i}\right){\ddot{q}}_{i}-2C_{i}\left(q_{i},{\dot{q}}_{i}\right)$ *is a skew symmetric matrix; in other words*,

$$x^{T}\left(\dot{M}\left(q\right)-2C\left(q,\dot{q}\right)\right)x=0,\;\forall x\in {\text{R}}^{N.n\times 1}$$

Property 3. *The inertia matrix M*(*q*) *is symmetric positive-definite for a manipulator with revolute joints, and has the following upper and lower bounds:*

$$0<\lambda_{min}\left(M\left(q\left(t\right)\right)\right)I\leq M\left(q\left(t\right)\right)\leq \lambda_{max}\left(M\left(q\left(t\right)\right)\right)I<\infty$$

*or equivalently*,

$$0<\frac{1}{\lambda_{max}}(M^{-1}(q(t))\;I\leq M^{-1}(q(t))\leq \frac{1}{\lambda_{min}}(M^{-1}(q))I<\infty$$

*where* λ_*i*_ *denotes the ith eigenvalue of a matrix, and I* ∈ *R*^*n*×*n*^ *is the identity matrix*.

Furthermore, the derivative of the inverse of a matrix can be calculated as:

$$\frac{d}{dt}\left\{M{\left(q\right)}^{-1}\right\}=-M{\left(q\right)}^{-1}\frac{d}{dt}\left\{M\left(q\right)\right\}M{\left(q\right)}^{-1}$$

Property 4. *The dynamics of the manipulator, written in (1) equation, can be parameterized linearly as*

$$M_{i}\left(q_{i}\right){\ddot{q}}_{i}+C_{i}\left(q_{i},{\dot{q}}_{i}\right){\dot{q}}_{i}+k_{i}q_{i}=\theta_{i}\left(q_{i},{\dot{q}}_{i}\right)Y_{i}+\tau_{c_{i}}-\tau_{h_{i}}$$

*in which, the matrix* Yi *is the regressor matrix including known robot signals, and* $\theta_{i}\left(q_{i},{\dot{q}}_{i}\right)$ *is the vector of unknown robot parameters (Cheah et al., 2006)*. τ_*h*_*i*__ *is the torque applied by the operator of the ith robot, and* $\tau_{c_{i}}\in {\text{R}}^{n\times 1}$ *is the control torque of the ith robot*.

Assumption 1. *(Deng, 2014) Based on the passivity assumption of human operators and the environment, there are positive constants* κ_*i*_ *such that for the ith operator, the passivity relation is*

$$\int_{0}^{t}\dot{q}{{\left(s\right)}_{i}}^{T}\tau_{h_{i}}\left(s\right)ds+\kappa_{i}\geq 0$$

*Summing the above equations for i* = 1, …, *N and rewriting in matrix form we have*

(2) $$\begin{array}{l} \int_{0}^{t}\dot{Q}{\left(s\right)}^{T}T_{H}\left(s\right)ds+\Upsilon \geq 0 \end{array}$$

*where* $Q_{n.N\times 1}={\left[q_{1}^{T}\cdots q_{N}^{T}\right]}^{T}$, $T_{H}={\left[\begin{array}{ccc} \tau_{h_{1}}^{T} & \cdots & \tau_{h_{N}}^{T} \end{array}\right]}^{T}$, *and* $\Upsilon =\overset{N}{\underset{i=1}{\sum }}\kappa_{i}$.

### 2.3. Some Definitions in Multi-Lateral Teleoperation Systems

In the following, some definitions that are useful for the rest of the paper are addressed.

Definition 1. ***Shared Environment** is a virtual collaborative environment that brings together users who are geographically distributed but connected via a network*.

Definition 2. ***Assistive/Resistive Rehabilitation: Assistive Rehabilitation** provides an assistant force for the users to complete the target movement. Conversely*, ***Resistive Rehabilitation** provides a resistant force against the movement. The proposed system in this paper, can provide the both phases, meaning that it can either help the user's movement in the target direction in assistive phase or constrain the direction of the user's movements, preventing deviations from the target trajectory in the resistive phase (Brewer et al., 2007)*.

Definition 3. *The term **Transparency** refers to the fact that if the operators feel they are directly interacting with the remote task, the teleoperation system would be completely transparent. Meaning that the operator's position (X*_*m*_*) can be exerted on the remote task while he/she simultaneously feels the force of the environment (F*_*s*_*)*.

Definition 4. *The term **Hierarchical Teleoperation** can be defined as an attempt to handle the problem of cooperative multi-lateral teleoperation systems by decomposing the problem of teleoperation into smaller subproblems and reassembling their solutions into a hierarchical structure. In this structure, the operators located in an upper layer command the weighted average of their forces/positions to the lower layer, and get the desired forces/positions from the operators in the lower layers*.

*In this structure, the operators (agents) at the master or slave sides may not connect directly together and can get/share the information indirectly from/to other operators via an intermediate operator*.

Definition 5. ***Multi-lateral Teleoperation** system is the system in which multiple robots interact with each other to perform a remote task in shared environments. So, these robots can manipulate an object in the shared virtual environment through an intervening tool or directly. In the multi-lateral teleoperation system, the information can flow between all sites. Depending on the number of channels used in the control architecture, this information can include position and/or force information. A multi-lateral teleoperation system comprises multiple robots as haptic interfaces for multiple operators*.

Definition 6. *The force sensed by the hand of the operator, in the teleoperation process is called **Sensed Force** in this literature. It is equal to* τ_*ext*_*i*__ *in (1)*.

### 2.4. Using MAS Framework for Multi-Lateral Teleoperation

In this subsection, a correspondence (mapping) between the multi-lateral teleoperation systems and MAS will be constituted. Due to this correspondence, the following consideration should be taken.

All the master robots in the teleoperation system are considered as leaders in the MAS, and all the slave robots are assumed as followers. Hence, the structure of cooperative teleoperation can be considered as the leader-follower scheme in the MAS. In addition, the masters' and slaves' positions must track each other. This objective is similar to the convergence of the positions of the agents in the MAS. Moreover, any latency in the communication channels is regarded as delays of the agent to agent connections in the MAS. One property of MAS is the synchronization, meaning that despite the limited connectivity between the neighbors the tracking objective is done if the spanning tree exists (Zuo et al., 2016). Based on this fact, in the proposed method, the tracking of positions in a multi-lateral teleoperation system is shown to be possible as long as the spanning tree still exists, even if some connections in the network are broken.

A graph of multi-agent system with network topology G is considered. In this topology, if the agent *i* cannot receive any information from agent *j*, then α_*ij*_ in the adjacency matrix will be chosen as zeros; otherwise, it will be a positive scalar related to the connection weight. The index of α_*ij*_ shows the value of connection weight from the *j*th agent to the *i*th agent. Theses values can be regarded as the “performance” or “interference” index in the related studies like Rohrer et al. (2002).

In this study, the position error for the *i*th agents is defined as $e_{i}\left(t\right)=\underset{j\in N_{i}}{\sum }\alpha_{ij}\left(q_{i}\left(t\right)-q_{j}\left(t\right)\right)$, and the torque effort for *i*th manipulator should contain the following terms as a function of position error:

(3) $$\begin{array}{l} {{\bar{\tau }}_{c}}_{i}\left(t\right)=-\underset{j\in N_{j}}{\sum }\alpha_{ji}{\bar{p}}_{i}e_{j}\left(t\right) \end{array}$$

where ${\bar{p}}_{i}\geq 0$ is a weight scalar. In section 3 it will be shown that the use of (3) as part of the control effort, helps to make the multilateral teleoperation system transparent.

Remark 3. *The term **Centralized Controller** refers to the original multi-variable controller, which is located in the main computer (consisting of the interacting local controllers), while the term **Decentralized Controller** refers to a set of controllers inside each individual operator, which can communicate with each other with a reduced number of interconnection links. Consequently, using decentralized controllers may help the stability and connectivity of the system even if some certain commutation links in the system are lost. Moreover, in the decentralized controller scheme, each part (agent) has its own local controller that helps the system's reliability*.

## 3. Multi-Lateral Teleoperation Based on Centralized Controller

For a multi-lateral teleoperation system, a new centralized controller based on centralized MAS is introduced in this section. So, this section is a reference for the next section about the MAS-based decentralized controllers.

Consider the non-linear dynamic equation given as (1) for the *n*-DOF manipulator robots. The *N* robots (agents) equation can be augmented together, based on the following definitions,

(4) $$\begin{array}{l} M\left(Q\left(t\right)\right)\ddot{Q}\left(t\right)+C\left(Q\left(t\right),\dot{Q}\left(t\right)\right)\dot{Q}\left(t\right)+G\left(Q\left(t\right)\right)\\ =-T_{Ext}\left(t\right)+T_{C}\left(t\right) \end{array}$$

in which

$$\begin{array}{l} M_{n.N\times n.N}=diag\left\{M_{1},M_{2},\ldots ,M_{N}\right\}\\ \;C_{n.N\times n.N}=diag\left\{C_{1},C_{2},\ldots ,C_{N}\right\}\\ \;G_{n.N\times 1}={\left[{G_{1}}^{T},{G_{2}}^{T},\ldots ,{G_{N}}^{T}\right]}^{T}\\ {T_{Ext}}_{\;n.N\times 1}={\left[\tau_{{ext}_{1}}^{T}\cdots \tau_{{ext}_{N}}^{T}\right]}^{T}\\ \;{T_{C}}_{n.N\times 1}={\left[\tau_{c_{1}}^{T}\cdots \tau_{c_{N}}^{T}\right]}^{T}\\ \;Q_{n.N\times 1}={\left[q_{1}^{T}\cdots q_{N}^{T}\right]}^{T} \end{array}$$

Property 5. *It is easy to show that Property 2 can be generalized to the augmented dynamics of the operators in (4). The augmented version of Property 2 is*

$$X^{T}\left(\dot{M}\left(Q\right)-2C\left(Q,\dot{Q}\right)\right)X=0\;\forall X\in {\text{R}}^{N.n\times 1}$$

Remark 4. *Consider the matrix* $\bar{P}=\text{diag}\left\{{\bar{p}}_{1},\ldots ,{\bar{p}}_{N}\right\}$ *and the following equation:*

$$P_{N.n\times N.n}={\bar{P}}_{N\times N}⊗I_{n\times n}$$

So, the following equation can directly be shown, based on the *Kronecker product* properties:

$${\left(L⊗I_{n\times n}\right)}^{T}P\left(L⊗I_{n\times n}\right)=\left(L^{T}\bar{P}L\right)⊗I_{n\times n}$$

*It is also straightforward to show that if a positive definite P is chosen, then* $\bar{P}$ *will be positive definite, too*.

The controller's augmented position error is described as:

(5) $$\begin{array}{l} E\left(t\right)={\left[e_{1}^{T},\ldots ,e_{N}^{T}\right]}^{T}={\left(L_{N\times N}⊗I_{n\times n}\right)}_{N.n\times N.n}.Q{\left(t\right)}_{N.n\times 1} \end{array}$$

where *e*_*i*_(*t*) is

(6) $$\begin{array}{l} e_{i}\left(t\right)=\left(L⊗I\right)Q\left(t\right) \end{array}$$

which is the position errors for the *i*th agent and its neighbors.

The controller is designed as

(7) $$\begin{array}{l} \tau_{c_{i}}\left(t\right)=g_{i}\left(q\left(t\right)\right)-\Gamma_{i}{\dot{q}}_{i}\left(t\right)+{\bar{\tau }}_{c_{i}}\left(t\right) \end{array}$$

in which ${\bar{\tau }}_{c_{i}}\left(t\right)$ is defined as (3). The augmented form of ${\bar{\tau }}_{c_{i}}\left(t\right)$ and τ_*c*_*i*__(*t*) is as follows:

(8) $$\begin{array}{l} T_{C}\left(t\right)=G\left(Q\right)-\Gamma_{N.n\times N.n}.\dot{Q}\left(t\right)+{\bar{T}}_{C}\left(t\right) \end{array}$$

(9) $$\begin{array}{l} {\bar{T}}_{C}\left(t\right)={\left(\left(L^{T}\bar{P}L\right)⊗I_{n\times n}\right)}_{N.n\times N.n}.Q_{N.n\times 1}\left(t\right) \end{array}$$

where Γ is the positive-definite damping factor of the system and is a positive definite matrix which can be chosen as

$$diag\left\{\Gamma_{1},\ldots ,\Gamma_{N}\right\}$$

**Figure 1:**
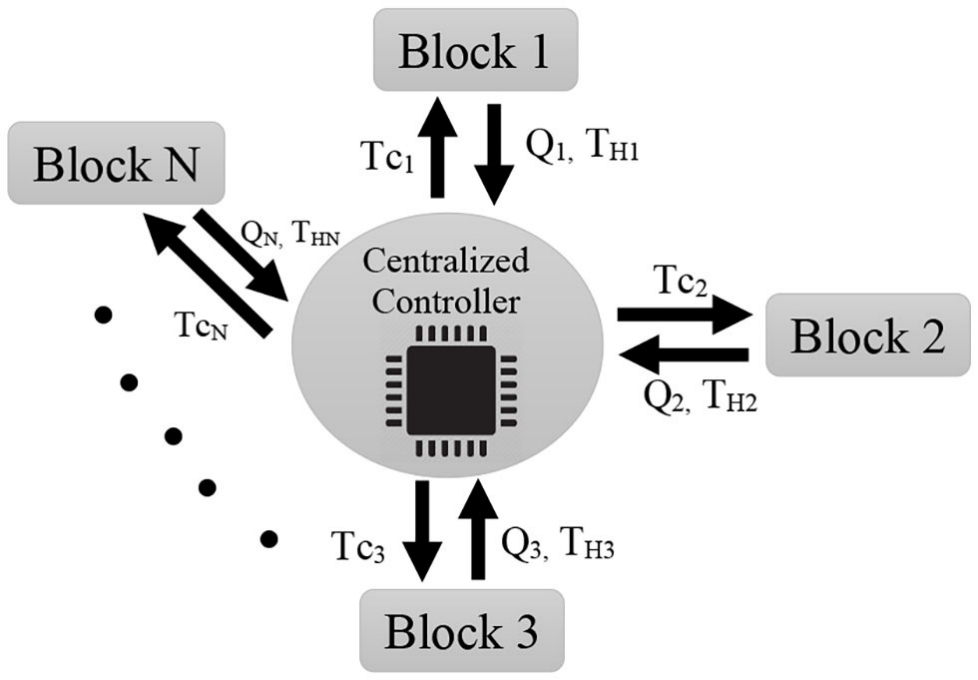
Centralized controller. Each block sends its position and sensed force to the central controller. The central controller calculates the control torque using (8) and sends its associated part to each individual block (i.e., {*T*_*C*1_, …, *T*_*CN*_}). As a drawback of the centralized controller, it is clear that if the centralized controller is damaged, the whole system will fail.

The idea of the centralized controller is depicted in Figure 1. Accordingly, the closed-loop equation of the system would results as follows

(10) $$\begin{array}{l} M\left(Q\left(t\right)\right)\ddot{Q}\left(t\right)+C\left(Q\left(t\right),\dot{Q}\left(t\right)\right)\dot{Q}\left(t\right)=-T_{H}\left(t\right)\\ +\left(\left(L^{T}\bar{P}L\right)⊗I_{n\times n}\right).Q_{N.n\times 1}\left(t\right)-\Gamma_{N.n\times N.n}.\dot{Q}\left(t\right) \end{array}$$

In the following part, the first result of the suggested controller is presented as a theorem.

Theorem 1. *If the augmented controller (8) is exerted on the multi-lateral teleoperation system (4), and considering the assumption 1, then the vectors of augmented joint velocity and acceleration* $\dot{Q}\left(t\right)$, $\ddot{Q}\left(t\right)$ *and the augmented joint position error* E(t) *will remain bounded for* α_*ij*_ ≥ 0.

*Proof*. Consider the Lyapunov candidate as the following scalar functionals:

$$\begin{array}{l} V_{1}\left(t\right)=\frac{1}{2}\overset{N}{\underset{i=1}{\sum }}{\dot{q}}^{T}M_{i}\left(q\left(t\right)\right)\dot{q}=\frac{1}{2}{\dot{Q}}^{T}M\left(Q\left(t\right)\right)\dot{Q}\\ V_{2}\left(t\right)=\frac{1}{2}\overset{N}{\underset{i=1}{\sum }}e_{i}{\left(t\right)}^{T}.p_{i}.e_{i}\left(t\right)=\frac{1}{2}E{{\left(t\right)}^{T}}_{1\times N.n}\;P_{N.n\times N.n}\\ \;\times E{\left(t\right)}_{N.n\times 1}\\ \;=Q^{T}\left({\left(L⊗I_{n\times n}\right)}^{T}P\left(L⊗I_{n\times n}\right)\right)Q\left(t\right)\\ \;=Q^{T}\left(\left(L^{T}\bar{P}L\right)⊗I_{n\times n}\right)Q\left(t\right)\\ V_{3}\left(t\right)\;=\int_{o}^{t}\dot{Q}{\left(s\right)}^{T}T_{H}\left(s\right)dt+\Upsilon \end{array}$$

So, by summing up *V*_*i*_*s* we have

(11) $$\begin{array}{l} V\left(t\right)=V_{1}\left(t\right)+V_{2}\left(t\right)+V_{3}\left(t\right) \end{array}$$

Subsequently,

(12) $$\begin{array}{l} \dot{V}\left(t\right)=\frac{1}{2}{\dot{Q}}^{T}\left(t\right)M\left(Q\left(t\right)\right)\ddot{Q}\left(t\right)+\frac{1}{2}{\dot{Q}}^{T}\left(t\right)\dot{M}\left(Q\left(t\right)\right)\dot{Q}\left(t\right)\\ \;+{\dot{Q}}^{T}\left(\left(L^{T}\bar{P}L\right)⊗I_{n\times n}\right)Q+{\dot{Q}}^{T}T_{H}\left(t\right) \end{array}$$

Using (8) and (9) results in

(13) $$\begin{array}{l} \dot{V}(t)={\dot{Q}}^{T}(-T_{H}+T_{C}-C\dot{Q}-G\\ \;+\frac{1}{2}\dot{M}(Q(t))\dot{Q})+{\dot{Q}}^{T}(\left(L^{T}\bar{P}L\right)⊗I_{n\times n})Q+{\dot{Q}}^{T}T_{H}\\ \;={\dot{Q}}^{T}(-T_{H}+T_{C}-C\dot{Q}-G)\\ \;={\dot{Q}}^{T}({\bar{T}}_{C}-(\left(L^{T}\bar{P}L\right)⊗I_{n\times n})Q)-{\dot{Q}}^{T}(t)\Gamma \dot{Q}(t)\\ \;=-\dot{Q^{T}}(t)\Gamma \dot{Q}(t)\leq 0 \end{array}$$

Thus, the positive scalar *V*(*t*) in (11) is non-increasing for any α_*ij*_ ≥ 0; it satisfies the boundedness of $\dot{Q}\left(t\right)$ and E(t).

(14) $$\begin{array}{l} \ddot{Q}\left(t\right)=M^{-1}\left(Q\left(t\right)\right)\{-\Gamma_{N.n\times N.n}\cdot \dot{Q}\left(t\right)\\ \;+{\left({\left(L^{T}\bar{P}L\right)}_{N\times N}⊗I_{n\times n}\right)}_{N.n\times N.n}Q\left(t\right)+C\left(Q,\dot{Q}\right)\dot{Q}-T_{H}\left(t\right)\} \end{array}$$

Using Equation (14), and property 1 and 3, it is easy to show that $\ddot{Q}\left(t\right)$ is bounded, too, which completes the proof.

    □

Remark 5. *It is easy to see from (10) that, at the steady-state* $\left(e.g.,\dot{Q}\left(t\right),\ddot{Q}\left(t\right)≃0\right)$, *the sensed force is as follows:*

(15) $$\begin{array}{l} T_{H}\left(\infty \right)=\left[\left(L^{T}\bar{P}L\right)⊗I_{n\times n}\right]Q\left(\infty \right) \end{array}$$

*The above-mentioned fact is utilized in section 6*.

Corollary 1. *In the multi-lateral teleoperation system with the same conditions as in Theorem 1 and working in free motion, i.e.*, τ_*h*_*i*__(*t*) = 0 *for i* = 1, …, *N* *(or equivalently* $T_{Ext}\left(t\right)=0$), *and the other assumptions as in Theorem 1, the absolute values of the position errors* (|*e*_*i*_(*t*)|) *and the joint velocities* ($\left|{\dot{q}}_{i}\left(t\right)\right|$) *asymptotically converge to zero*.

*Proof*. Integrating (12), and noting that V(Q(t))≥0 in relation (11), results in

$$0\geq \int_{0}^{t}\left(-{\dot{Q}}^{T}\left(s\right)\Gamma \dot{Q}\left(s\right)\right)ds=V\left(t\right)-V\left(0\right)\geq -V\left(0\right)$$

Therefore, $0\leq \lambda_{\min}\left(\Gamma \right){\left\|\dot{Q}\left(t\right)\right\|}_{2}^{2}\leq V\left(0\right)$. So, $\dot{Q}\in {\text{L}}_{2}$, which yields in ${\dot{q}}_{i}\left(t\right)\in {\text{L}}_{2},\;\forall i\in \left\{1,\ldots ,N\right\}$. Furthermore, with a lower-bounded decreasing function V(Q(t)), it is concluded that

$$E{\left(t\right)}_{N.n\times 1}={\left(L_{N\times N}⊗I_{n\times n}\right)}_{N.n\times N.n}.Q{\left(t\right)}_{N.n\times 1}\in {\text{L}}_{\infty }$$

And on the other side,

(16) $$\begin{array}{l} \overset{..}{Q}(t)=M^{-1}(Q(t))(-\Gamma_{N.n\times N.n}\;.\;\dot{Q}(t)\\ \;+{\left({\left(L^{T}\bar{P}L\right)}_{N\times N}⊗I_{n\times n}\right)}_{N.n\times N.n}Q(t)+C(Q,\dot{Q})\dot{Q}) \end{array}$$

So, from (16), it is obvious that $\ddot{Q}\left(t\right)\in {\text{L}}_{\infty }$, which yields in $\ddot{q_{i}}\left(t\right)\in {\text{L}}_{\infty },\;\forall i\in \left\{1,\ldots ,N\right\}$. Up to now, it was shown that $\dot{q_{i}}\left(t\right)\in L_{\infty }\cap L_{2}$, and $\ddot{q}\left(t\right)\in L_{\infty }$ for all *i* ∈ {1, …, *N*}. Accordingly, using the Barbalat's lemma, ${\dot{q}}_{i}\left(\infty \right)\to 0$. Hence, from (6), *e*_*i*_(*t*) converge to zero, asymptotically.

On the other hand, from (16) Equation (17) is concluded as follows:

(17) $$\begin{array}{l} \overset{\ldots }{Q}(t)=\frac{d}{dt}\left\{M^{-1}(Q(t))\right\}(-\Gamma \;.\;\dot{Q}(t)\\ \;+\left[\left(L^{T}\bar{P}L\right)⊗I_{n\times n}\right]Q(t)+C(Q,\dot{Q})\dot{Q})\\ \;+M^{-1}(Q)\frac{d}{dt}(-\Gamma \;.\;\dot{Q}(t)\;\\ \;+(\left(L^{T}\bar{P}L\right)⊗I_{n\times n})Q(t)+C(Q,\dot{Q})\dot{Q}) \end{array}$$

Based on the properties I and III, $\frac{d}{dt}\left\{M^{-1}\left(Q\right)\right\}$ is bounded. Therefore, it can be concluded that (17) is bounded or equivalently $\overset{\mathrm{...}}{Q}\left(t\right)\in {\text{L}}_{\infty }$. So, ${\dot{q}}_{i}\left(t\right)\in {\text{L}}_{\infty },\;\forall i\in \left\{1,\ldots ,N\right\}$. Therefore, $\ddot{Q}\left(t\right)$ is continuous in time. Hence, using Barbalet's lemma, $\ddot{Q}\left(t\right)\to 0$.

Accordingly, from (10), $T_{H}\left(\infty \right)={\left(L\bar{P}⊗I\right)}^{T}E\left(\infty \right)\to 0$. Consequently, the operators' sensed forces asymptotically converge to zero.

    □

Remark 6. *In Theorem 1 and Corollary 1, it was shown that by certain control efforts, the position errors could be reduced. On the other hand, by Remark 5 the hands' sensed force of the operators can be adjusted in the steady-state. So, the transparency of the system defined in Definition 3 can be achieved*.

## 4. Uncertain Dynamics in the Environment and the Manipulators

Uncertainty in the dynamics of the manipulators is discussed in this section. Consider the augmented dynamics of the manipulators as before mentioned:

(18) $$\begin{array}{l} M\left(Q\left(t\right)\right)\ddot{Q}\left(t\right)+C\left(Q\left(t\right),\dot{Q}\left(t\right)\right)\dot{Q}\left(t\right)+G\left(Q\left(t\right)\right)\\ =-T_{H}\left(t\right)+T_{C}\left(t\right) \end{array}$$

The controller $T_{C}\left(t\right)$ is now defined as

(19) $$\begin{array}{l} T_{C}\left(t\right)=\hat{M}\left(Q\left(t\right)\right)\dot{V}\left(t\right)+\hat{C}\left(Q\left(t\right),\dot{Q}\left(t\right)\right)V\left(t\right)\\ \;+\hat{G}\left(t\right)\left(Q\left(t\right)\right)-KR\left(t\right)+{\bar{T}}_{C}\left(t\right)\\ \;=Y\left(t\right)\hat{\Theta }\left(t\right)-KR\left(t\right)+{\bar{T}}_{C}\left(t\right) \end{array}$$

while ${\bar{T}}_{C}\left(t\right)$ is defined as

(20) $$\begin{array}{l} {\bar{T}}_{C}\left(t\right)=-\left(\left(L^{T}\bar{P}\right)⊗I_{n\times n}\right)E\left(t\right) \end{array}$$

The adaptation law is regarded as

(21) $$\begin{array}{l} \dot{\hat{\Theta }}=\Omega^{-T}Y^{T}\left(t\right)R\left(t\right) \end{array}$$

in which Ω is positive definite matrix. We can re-write the controller (19) as

$$\begin{array}{l} T_{C}\left(t\right)=\hat{M}\left(Q\right)\dot{V}\left(t\right)+\hat{C}\left(t\right)\left(Q\left(t\right),\dot{Q}\left(t\right)\right)V\left(t\right)+\hat{G\left(t\right)}\left(Q\left(t\right)\right)\\ \pm \left(M\left(Q\right)\dot{V}\left(t\right)+C\left(Q,\dot{Q}\right)V\left(t\right)+G\left(Q\right)\right)-KR\left(t\right)+{\bar{T}}_{C}\left(t\right)\\ \;=Y\hat{\Theta }\pm Y\Theta -KR\left(t\right)+{\bar{T}}_{C}\left(t\right) \end{array}$$

The symbol ± means that YΘ is added and subtracted to and from the equation. Subsequently, using the controller (19), we can re-arrange the closed-loop dynamics of the system (18) as

$$\begin{array}{l} M\left(Q\right)\left(\ddot{Q}-\dot{V}\right)+C\left(Q,\dot{Q}\right)\left(\dot{Q}-V\right)+K.R\left(t\right)\\ =\left(\hat{M}\left(Q\right)-M\left(Q\right)\right)\dot{V}\left(t\right)+\left(\hat{C}\left(Q,\dot{Q}\right)-C\left(Q,\dot{Q}\right)\right)V\left(t\right)\\ \;+\left(\hat{G}-G\right)+{\bar{T}}_{C}\left(t\right)-T_{H}\left(t\right) \end{array}$$

yielding

(22) $$\begin{array}{l} M\left(Q\right)\dot{R}\left(t\right)=Y\left(t\right)\tilde{\Theta }\left(t\right)+{\bar{T}}_{C}\left(t\right)-C\left(Q\left(t\right),\dot{Q}\right)R\left(t\right)\\ \;-K.R\left(t\right)-T_{H}\left(t\right) \end{array}$$

Therefore, the parameter R(t) is chosen based on (22) as

(23) $$\begin{array}{l} R\left(t\right)=\dot{Q}\left(t\right)-V\left(t\right) \end{array}$$

R(t) is inherently a low-pass filter. So, this filter can be considered as follows

(24) $$\begin{array}{l} R\left(t\right)=\dot{Q}\left(t\right)+\lambda \left(L⊗I_{n\times n}\right)Q\left(t\right) \end{array}$$

meaning that

(25) $$\begin{array}{l} V\left(t\right)=-\lambda \left(L⊗I_{n\times n}\right)Q\left(t\right) \end{array}$$

Assumption 2. *The human operators' hand force follows the below equation*

$$T_{H}\left(t\right)=\kappa_{0}\left(t\right)+\kappa_{1}R\left(t\right)$$

*in which* R *is as defined in (24) and*

$$\kappa_{0}\left(t\right)={\left[\kappa_{0_{1}}^{T}\left(t\right),\ldots ,\kappa_{0_{N}}^{T}\left(t\right)\right]}^{T}\kappa_{1}\left(t\right)={\left[\kappa_{1_{1}}^{T}\left(t\right),\ldots ,\kappa_{1_{N}}^{T}\left(t\right)\right]}^{T}.$$

*Moreover, it is assumed that every element of* κ_0_ *and* κ_1_ *are bounded. Furthermore, note that* κ_0_(*t*) *can be argued as a pure muscular force of the operators' hand, which is obviously bounded*.

Theorem 2. *By Assumption 2 on the operators hand force, in the multi-lateral teleoperation system with the uncertain augmented dynamics (18), and the controllers (19), (21), (23), and (25) with damping coefficient* Γ *as a positive-definite matrix and* α_*ij*_ ≥ 0, *the augmented joint position error* E(t) *will ultimately remain bounded*.

*Proof*. Consider the following Lyapunov functionals

(26) $$\begin{array}{l} V_{1}\left(t\right)=\frac{1}{2}R^{T}\left(t\right)MR\left(t\right)\\ V_{2}\left(t\right)=\frac{1}{2}{\tilde{\Theta }}^{T}\left(t\right)\Omega \tilde{\Theta }\left(t\right)\\ V_{3}\left(t\right)=\frac{1}{2}E^{T}\left(t\right)PE\left(t\right) \end{array}$$

The summation of *V*_*i*_s are as

$$V\left(t\right)=V_{1}\left(t\right)+V_{2}\left(t\right)+V_{3}\left(t\right)$$

Then, we have

(27) $$\begin{array}{l} {\dot{V}}_{1}\left(t\right)=R^{T}\left(t\right)\left(Y\tilde{\Theta }\left(t\right)+{\bar{T}}_{C}\left(t\right)-CR\left(t\right)-KR\left(t\right)\right)\\ {\dot{V}}_{2}\left(t\right)={\dot{\tilde{\Theta }}}^{T}\left(t\right)\Omega \tilde{\Theta }\left(t\right)\\ {\dot{V}}_{3}\left(t\right)={\dot{E}}^{T}\left(t\right)PE\left(t\right) \end{array}$$

Using (24) inside *V*_4_(*t*), we have,

$$\begin{array}{l} {\dot{V}}_{3}\left(t\right)=\left(R^{T}\left(t\right)-\lambda Q^{T}\left(t\right)\left(L⊗I_{n\times n}\right)\right){\left(L⊗I_{n\times n}\right)}^{T}PE\left(t\right)\\ \;=R^{T}\left(t\right){\left(L⊗I_{n\times n}\right)}^{T}PE\left(t\right)\\ \;-\lambda Q^{T}\left(t\right)\left(\left(L⊗I_{n\times n}\right){\left(L⊗I_{n\times n}\right)}^{T}P\right)E\left(t\right)\\ \;=R^{T}\left(t\right){\left(L⊗I_{n\times n}\right)}^{T}PE\left(t\right)\\ \;-\lambda E^{T}\left(t\right)\left(\left(L^{T}⊗I_{n\times n}\right)P\right)E\left(t\right) \end{array}$$

So, the result of $\dot{V}\left(t\right)$ would be as follows,

(28) $$\begin{array}{l} \dot{V}\left(t\right)=-R^{T}\left(t\right)KR\left(t\right)-R^{T}\left(t\right)T_{H}\left(t\right)\\ \;-\lambda E^{T}\left(t\right)\left(\underset{\text{Positive }S-\text{Definite}}{\underset{︸}{\left(L^{T}⊗I_{n\times n}\right)P}}\right)E\left(t\right)\leq 0 \end{array}$$

by using *Assumption 2*, the result can be written as

(29) $$\begin{array}{l} \dot{V}\left(t\right)=-R^{T}\left(t\right)\left(\underset{\Xi }{\underset{︸}{K+\kappa_{1}}}\right)R\left(t\right)-R^{T}\left(t\right)\kappa_{0}\left(t\right)\\ \;-\lambda E^{T}\left(t\right)\left(\left(L^{T}⊗I_{n\times n}\right)P\right)E\left(t\right) \end{array}$$

Using the fact that Ξ = *K* + κ_1_ is positive definite and symmetric,

$$\begin{array}{l} \dot{V}\left(t\right)=-\frac{1}{2}R^{T}\left(t\right)\Xi R\left(t\right)-\frac{1}{2}\left[R^{T}\left(t\right)\left(\theta \Xi \right)R\left(t\right)+2R^{T}\left(t\right)\kappa_{0}\left(t\right)\right]\\ \;-\frac{1}{2}R^{T}\left(t\right)\left(\left(1-\theta \right)\Xi \right)R\left(t\right)-\lambda E^{T}\left(t\right)\left(\left(L^{T}⊗I_{n\times n}\right)P\right)E\left(t\right)\\ \;\leq -\frac{1}{2}R^{T}\left(t\right)\Xi R\left(t\right)-\lambda E^{T}\left(t\right)\left(\left(L^{T}⊗I_{n\times n}\right)P\right)E\left(t\right)\\ \;-\frac{1}{2}R^{T}\left(t\right)\left(\left(1-\theta \right)\Xi \right)R\left(t\right)+\frac{1}{2}\kappa_{0}^{T}{\left(\theta \Xi \right)}^{-1}\kappa_{0} \end{array}$$

So,

$$\begin{array}{l} \dot{V}\left(t\right)=-\frac{1}{2}R^{T}\left(t\right)\Xi R\left(t\right)-\frac{1}{2}\left[R^{T}\left(t\right)\left(\theta \Xi \right)R\left(t\right)+2R^{T}\left(t\right)\kappa_{0}\left(t\right)\right]\\ \;-\frac{1}{2}R^{T}\left(t\right)\left(\left(1-\theta \right)\Xi \right)R\left(t\right)-\lambda E^{T}\left(t\right)\left(\left(L^{T}⊗I_{n\times n}\right)P\right)E\left(t\right)\\ \;\leq -\frac{1}{2}R^{T}\left(t\right)\Xi R\left(t\right)-\lambda E^{T}\left(t\right)\left(\left(L^{T}⊗I_{n\times n}\right)P\right)E\left(t\right)\\ \;-\frac{1}{2}R^{T}\left(t\right)\left(\left(1-\theta \right)\Xi \right)R\left(t\right)+\frac{1}{2}\kappa_{0}^{T}{\left(\theta \Xi \right)}^{-1}\kappa_{0} \end{array}$$

$\left(L^{T}⊗I_{n\times n}\right)P$ is positive semi-definite, therefore

$$\dot{V}\left(t\right)\leq -\frac{1}{2}{\left\|R\right\|}^{2}\lambda_{\min}\left(\Xi \right)\left(1-\theta \right)+\frac{1}{2}{\left\|{\bar{\kappa }}_{0}\right\|}^{2}\lambda_{\min}\left(\Xi \right)\theta$$

On the other hand, if we choose Ω as follows

$$\Omega =\left\{R|\|R\|<\frac{1}{\lambda_{\min}\left(\Xi \right)\sqrt{\left(1-\theta \right)\theta }}\|{\bar{\kappa }}_{0}\|,0<\theta <1\right\}$$

then, outside the closed set Ω, $\dot{V}\left(t\right)$ is negative or zero. Therefore, E(t), $\tilde{\Theta }\left(t\right)$, and R(t) are UUB.

Considering the closed-loop dynamic (22), the fact R(t), $\tilde{\Theta }\left(t\right)$, and $E\left(t\right),\kappa_{0}\in {\text{L}}_{\infty }$, and it is concluded that $\dot{R}\left(t\right)\in {\text{L}}_{\infty }$. Moreover, from (24) it is concluded that

$$\dot{R}\left(t\right)=\ddot{Q}\left(t\right)+\lambda \left(L⊗{\text{I}}_{n}\right)\dot{Q}\left(t\right)$$

So, using the fact that $\dot{Q}\text{,}\dot{R}\left(t\right)\in {\text{L}}_{\infty }$, it is easy to show that $\ddot{Q}\in {\text{L}}_{\infty }$, which completes the proof.

    □

Remark 7. *Non-Passive Operators: If Assumption 2 holds and if the parameters* κ_0_ *or* κ_1_ *are negative; in other words, the operators are not passive, then the system is stable if K* + κ_1_ *still remain positive. According to non-passivity of the operators, the value of* κ_0_(*t*) *and* κ_1_ *may be negative (Chopra et al., 2008)*.

Assumption 3. *Pre-filtered passivity: A condition can be defined on the passivity filter as follows*

$$\int_{0}^{t}{r_{i}}^{T}\left(s\right)\tau_{h_{i}}\left(s\right)ds+\kappa_{i}\geq 0$$

**Figure 2:**
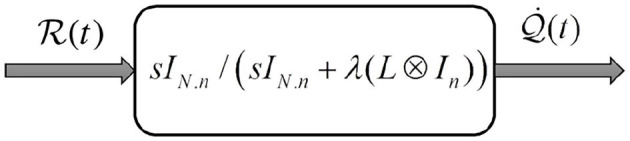
Pre-filtered passivity. This figure shows a multi-variable filter made of the passive filter. The division sign (forward slash) means that the left matrix is multiplied by the inverse of the right matrix.

*This condition is similar to assumption 1, however, the velocity signal is replaced with the pre-filtered passivity of the velocity signal as depicted in Figure 2 (Sharifi et al., 2017)*.

Theorem 3. *Assuming that the operators and the environment are pre-filtered passive as defined in assumption 3, in the multi-lateral teleoperation system with the uncertain augmented dynamics (18), and the controllers (19), (23), and (25), beside adaptation law (21), the augmented joint position error* E(t) *goes to zero asymptotically*.

*Proof*. Consider the following Lyapunov functionals as in (26) in addition to *V*_4_(*t*) defined in the following

$$V_{4}\left(t\right)=\left(\int_{0}^{t}R^{T}\left(t\right)T_{H}\left(t\right)dt+\Upsilon \right)$$

The Lyapunov function can be achieved by adding *V*_*i*_(*t*) where *i* ∈ 1, .., 4 as (30)

(30) $$\begin{array}{l} V\left(t\right)=V_{1}\left(t\right)+V_{2}\left(t\right)+V_{3}\left(t\right)+V_{4}\left(t\right) \end{array}$$

Moreover,

(31) $$\begin{array}{l} {\dot{V}}_{4}\left(t\right)=R^{T}\left(t\right)T_{H}\left(t\right) \end{array}$$

thus,

(32) $$\begin{array}{l} \dot{V}\left(t\right)=-R^{T}\left(t\right)KR\left(t\right)\\ \;-\lambda E^{T}\left(t\right)\left(\underset{\text{Positive Definite}}{\underset{︸}{\left(L^{T}⊗I_{n\times n}\right)P}}\right)E\left(t\right)\leq 0 \end{array}$$

Consequently, $\tilde{\Theta }\left(t\right)$, E(t), and $R\left(t\right)\in {\text{L}}_{\infty }$. So, from (23), $\dot{Q}\left(t\right)\in {\text{L}}_{\infty }$. Considering $\left(L^{T}⊗I_{n\times n}\right)P=\Upsilon$ and integrating (32), we have

$$\begin{array}{l} \int_{0}^{t}\left(-R^{T}\left(s\right)KR\left(s\right)\right)ds+\int_{0}^{t}\left(-E^{T}\left(s\right)\Upsilon E\left(s\right)\right)ds\\ =V\left(t\right)-V\left(0\right)\geq -V\left(0\right) \end{array}$$

Therefore,

$$0\leq \int_{0}^{t}\left(R^{T}\left(s\right)KR\left(s\right)\right)ds+\int_{0}^{t}\left(E^{T}\left(s\right)\Upsilon E\left(s\right)\right)ds\leq V\left(0\right)$$

Given 0 ≤ λ_*min*_(*K*) *I* ≤ *K* and 0 ≤ λ_*min*_(Υ) *I* ≤ Υ, it can be concluded that

$$0\leq \lambda_{\min}\left(K\right){\left\|R\left(t\right)\right\|}_{2}^{2}+\lambda_{\min}\left(\Upsilon \right){\left\|E\left(t\right)\right\|}_{2}^{2}\leq V\left(0\right)$$

Thus, R(t) and E(t) ∈ **L**_2_. Therefore, based on Barbalat's Lemma, the parameter E(t) converge to zero asymptotically.

    □

## 5. Decentralized Controller for Uncertain Systems in Presence of Varying Time Delay

**Figure 3:**
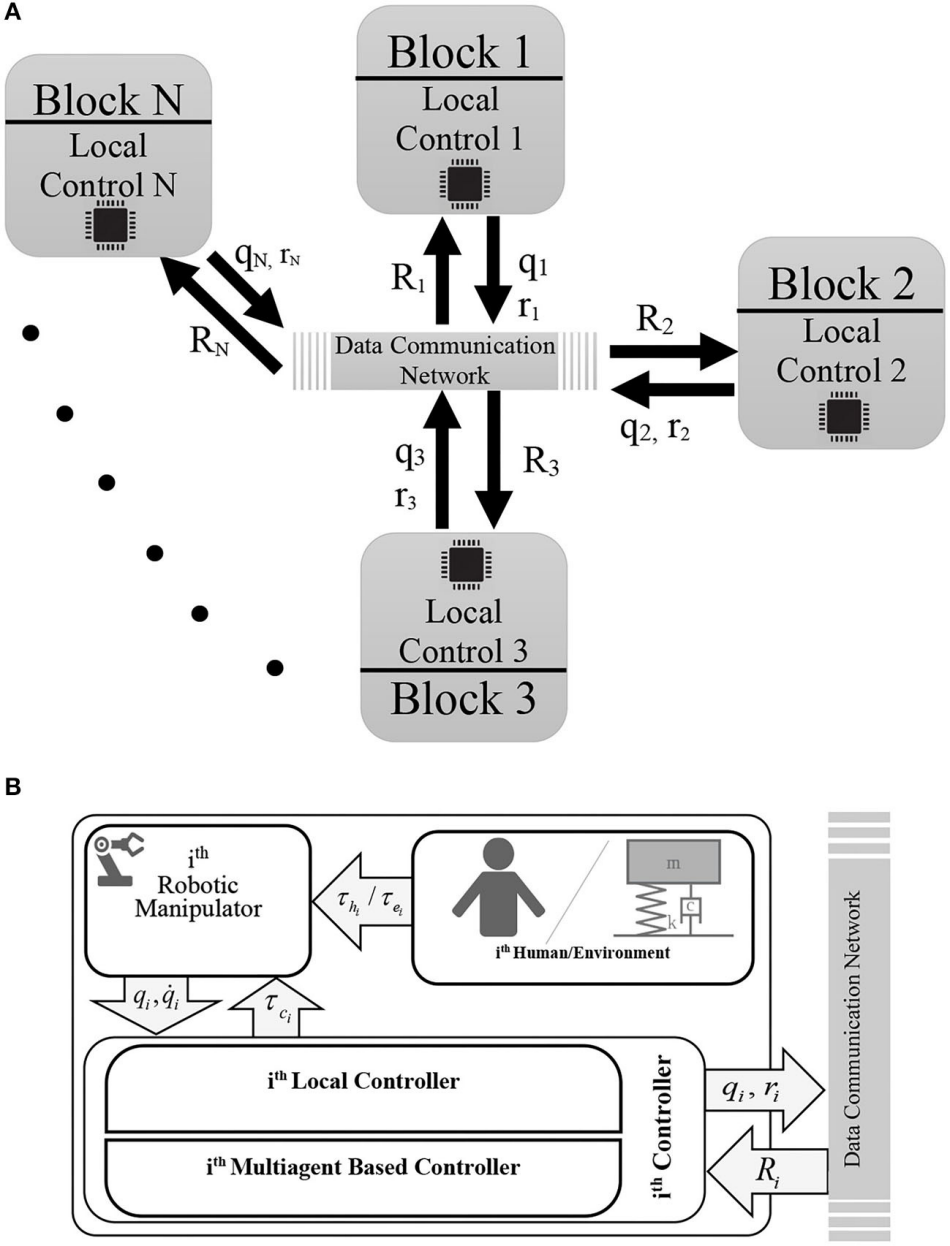
Diagram of the proposed method for decentralized controller. Part **(A)** shows the general overview of the decentralized controller. Part **(B)** depicts the inside of each block of the part **(A)**. In this diagram, the schematic of the local controller (35) is depicted. This controller consists of a local controller plus the multiagent based controller (37). To implement the latter one, the vector *R*_*i*_ is received from the adjacent agents of the *i*th manipulator, containing their intermediate variables *r*_*j*_ (j∈{A(i)}). On the other hand, the information about the *i*th position (*q*_*i*_) and the *i*th intermediate variable (*r*_*i*_) related to the *i*th robot itself, are shared with the neighbors via the data communication network.

In this section, the intelligence of each agent in the MAS is utilized in the concept of multi-lateral teleoperation systems, which were introduced in previous sections. Each operator works as an agent in MAS, and the local controller on each operator helps to synchronize positions and forces in the overall network based on *Definition 3*. These local controllers help to minimize the connection links, while minimizing the defective effects of varying time delays. There is no need to have a full connection between operators to set the multi-lateral teleoperation system (Figure 3). The only thing to have full control over the system is to have a spanning tree in the graph of the system (Su and Lin, 2016).

Moreover, it is shown in the rest part of this section that the proposed local controller can overcome uncertainty in the environment and the operator, while having time communication delays.

Assumption 4. *The delays which exist between the communication links of the operators, can be arbitrary and unknown, while its derivative should be bounded with a known upper-bound* ψ *of* ${\dot{\tau }}_{ji}\left(t\right)$, *i.e.*,

(33) $$\begin{array}{l} {\dot{\tau }}_{ji}\left(t\right)<\psi \end{array}$$

*Because of the causality of the delay, the derivative of the delay is considered to be less than unity, i.e.*, ψ ≤ 1.

The non-linear uncertain dynamics of the *i*th operator are as follows,

(34) $$\begin{array}{c} M_{i}\left(q_{i}\right){\ddot{q}}_{i}+C_{i}\left(q_{i},{\dot{q}}_{i}\right){\dot{q}}_{i}+g_{i}\left(q_{i}\right)=-\tau_{h_{i}}+\tau_{c_{i}} \end{array}$$

Note that, the parameters $q_{i},{\dot{q}}_{i},{\ddot{q}}_{i},\tau_{h_{i}},\tau_{c_{i}}$ are functions of time; however, for the sake of simplicity, the time parameter (t) is not written. In this part, because of time delays, the simple form of the augmented system (4) is not usable. So, the equation of each agent is written separately and integrated together. Moreover, the control law is chosen as

(35) $$\begin{array}{l} \tau_{c_{i}}={\hat{M}}_{i}(q_{i}){\dot{\upsilon }}_{i}+{\hat{C}}_{i}(q_{i},{\dot{q}}_{i})\upsilon_{i}+{\hat{g}}_{i}(q_{i})\\ \;+{\bar{\tau }}_{c_{i}}(M_{i}(q_{i}){\dot{\upsilon }}_{i}+C_{i}(q_{i},{\dot{q}}_{i})\upsilon_{i}\\ \;+g_{i}(q_{i}))-(M_{i}(q_{i}){\dot{\upsilon }}_{i}+C_{i}(q_{i},{\dot{q}}_{i})\upsilon_{i}+g_{i}(q_{i}))-k_{i}r_{i}\\ \;={\hat{\theta }}_{i}(q_{i},{\dot{q}}_{i})Y_{i}(t)-k_{i}r_{i}(t)+{\bar{\tau }}_{c_{i}} \end{array}$$

in which

(36) $$\begin{array}{l} k_{i}>\gamma_{i}\psi \underset{j\in N_{i}}{\sum }\alpha_{ji} \end{array}$$

Note that $\underset{j\in N_{i}}{\sum }\alpha_{ji}\leq N$. Furthermore, ${\bar{\tau }}_{c_{i}}$ is chosen as

(37) $$\begin{array}{l} {\bar{\tau }}_{c_{i}}=\frac{1}{2}\gamma_{i}\underset{j\in N_{i}}{\sum }\alpha_{ji}\left(-r_{i}+r_{j}\left(t-\tau_{ji}\left(t\right)\right)\right) \end{array}$$

in which *r*_*j*_(*t* − τ_*ji*_(*t*)) is received from the *j*th operator and γ_*i*_ is the *i*th element of the vector γ, which is the left eigen-vector of the Laplacian matrix according to zero eigenvalue of Laplacian matrix (see *Remark 2*).

So, the controller is consisted of two parts, the local controller [${\hat{\theta }}_{i}\left(q_{i},{\dot{q}}_{i}\right)Y_{i}\left(t\right)-k_{i}r_{i}\left(t\right)$] and the multiagent part (${\bar{\tau }}_{c_{i}}$). In addition *r*_*i*_(*t*) and υ_*i*_(*t*) are intermediate variables and are defined as $r_{i}\left(t\right)={\dot{q}}_{i}\left(t\right)-\upsilon_{i}\left(t\right)$ and υ_*i*_(*t*) = −λ*e*_*i*_(*t*). Hence,

(38) $$\begin{array}{l} r_{i}\left(t\right)={\dot{q}}_{i}\left(t\right)+\lambda e_{i}\left(t\right) \end{array}$$

consequently, (38) is a passive filter, containing the encoded data about the force/position errors. So, the closed loop system becomes

(39) $$\begin{array}{l} M_{i}\left(q_{i}\right){\dot{r}}_{i}+C_{i}\left(q_{i},{\dot{q}}_{i}\right)r_{i}+k_{i}r_{i}={\tilde{\theta }}_{i}\left(q_{i},{\dot{q}}_{i}\right)Y_{i}+{\bar{\tau }}_{c_{i}}-\tau_{h_{i}} \end{array}$$

or, equivalently:

(40) $$\begin{array}{l} M_{i}\left(q_{i}\right){\dot{r}}_{i}=-C_{i}\left(q_{i},{\dot{q}}_{i}\right)r_{i}\left(t\right)-k_{i}r_{i}\left(t\right)\\ \;+{\tilde{\theta }}_{i}\left(q_{i},{\dot{q}}_{i}\right)Y_{i}\left(t\right)+{\bar{\tau }}_{c_{i}}-\tau_{h_{i}} \end{array}$$

Furthermore, the adaptation law is considered as follows,

(41) $$\begin{array}{l} {\dot{\hat{\theta }}}_{i}={\Omega_{i}}^{-T}Y_{i}^{T}r_{i} \end{array}$$

in which, Ω_*i*_ is a positive definite matrix.

Theorem 4. *Consider a group of multi-lateral teleoperation systems, consisting of *N* manipulators with *n* degrees of freedom, with dynamical Equation (34), and control inputs (35), (37), and (41) with assumptions (33) and (36), then the synchronization error converges to zero asymptotically*.

*Proof*. Choosing the following Lyapunov candidate

$$\begin{array}{l} V(\xi_{t})=\frac{1}{2}\sum_{i\in N}\gamma_{i}(r_{i}^{T}M_{i}r_{i}+\sum_{j}{\tilde{\theta }}_{ij}^{T}\Omega_{i}{\tilde{\theta }}_{ij}-k_{i}r_{i}\\ \;+\int_{0}^{t}r_{i}{\;}^{T}\tau_{hi}dt+\kappa_{i}+\sum_{j\in N_{i}}\alpha_{ji}\int_{t-\tau_{ji}}^{t}r_{i}^{T}(s)r_{i}(s)ds) \end{array}$$

the derivative is

V˙(ξt)=12∑i∈Nγi(riTMir˙i+riTM˙iri+θ∼˙iTΩiθ˜i+ri Tτhi                 +∑j∈Niαji(rjTrj−rjT(t−τji(t))rj(t−τji(t))                +τ˙ji(t)rjT(t−τji(t))rj(t−τji(t)))

Equivalently, using (40) we have

(42) V˙(ξt)=12∑i∈Nγi(riT(−Ci(qi,q˙i)ri−kiri+Yiθ˜i(qi,q˙i)                +τ¯ci−τhi)+12γiriTM˙iri+12γiθ∼˙iTΩiθ˜i+12γiri Tτhi                 +12γi∑j∈Niαji(rjTrj−rjT(t−τji)rj(t−τji)               +τ˙ji(t)rjT(t−τji(t))rj(t−τji(t)))          =12∑i∈Nγi(−riTkiri+(riTYiθ˜i(qi,q˙i)+θ∼˙iTΩiθ˜i)             +riTτ¯ci+∑j∈Niαji(rjTrj−rjT(t−τji(t))rj(t−τji(t))           +τ˙ji(t)rjT(t−τji(t))rj(t−τji(t)))

It should be noted that,

(43) $$\begin{array}{l} \sum_{i\in N}r_{i}^{T}{\bar{\tau }}_{c_{i}}\\ \;+\frac{1}{2}\sum_{i\in N}\gamma_{i}\sum_{j\in N_{i}}\alpha_{ji}(r_{j}^{T}r_{j}-r_{j}^{T}(t-\tau_{ji}(t))r_{j}(t-\tau_{ji}(t))\\ \;+{\dot{\tau }}_{ji}(t)r_{j}^{T}(t-\tau_{ji}(t))r_{j}(t-\tau_{ji}(t)))\\ \;=\sum_{i\in N}\gamma_{i}\sum_{j\in N_{i}}\alpha_{ij}r_{i}^{T}\left(\left(r_{j}^{T}(t-\tau_{ji})-r_{i}(t\right)\right)\\ \;+\frac{1}{2}\sum_{i\in N}\gamma_{i}\sum_{j\in N_{i}}\alpha_{ji}(r_{j}^{T}r_{j}-r_{j}^{T}(t-\tau_{ji})r_{j}(t-\tau_{ji})\\ \;+{\dot{\tau }}_{ji}(t)r_{j}^{T}(t-\tau_{ji}(t))r_{j}(t-\tau_{ji}(t)))\\ \;=-\frac{1}{2}\sum_{i\in N}\gamma_{i}\sum_{j\in N_{i}}\alpha_{ji}(\left(r_{j}^{T}(t-\tau_{ji})r_{j}(t-\tau_{ji}\right)\\ \;-2r_{i}^{T}r_{j}(t-\tau_{ji})+r_{i}^{T}r_{j})\\ \;-\frac{1}{2}\sum_{i\in N}\gamma_{i}\sum_{j\in N_{i}}\alpha_{ji}(r_{i}^{T}r_{i}-r_{j}^{T}r_{j}\\ \;+{\dot{\tau }}_{ji}(t)r_{j}^{T}(t-\tau_{ji}(t))r_{j}(t-\tau_{ji}(t)))\\ =-\frac{1}{2}\sum_{i\in N}\gamma_{i}\sum_{j\in N_{i}}\alpha_{ji}\left({\left(.\right)}^{T}(\underset{{\dot{\epsilon }}_{ij}+\lambda \epsilon_{ij}}{\underset{︸}{r_{j}(t-\tau_{ji})-r_{i}}})\right)\\ \;-\frac{1}{2}\sum_{i\in N}\gamma_{i}\sum_{j\in N_{i}}\alpha_{ji}(r_{i}^{T}r_{i}-r_{j}^{T}r_{j}\\ \;+{\dot{\tau }}_{ji}(t)r_{j}^{T}(t-\tau_{ji}(t))r_{j}(t-\tau_{ji}(t)))\\ =-\frac{1}{2}\sum_{i\in N}\gamma_{i}\sum_{j\in N_{i}}\alpha_{ji}{\left(.\right)}^{T}\left({\dot{\epsilon }}_{ij}+\lambda \epsilon_{ij}\right)-\;\\ \;\frac{1}{2}\sum_{j\in N}\gamma_{i}\sum_{j\in N_{i}}\alpha_{ji}(r_{i}^{T}r_{i})\\ \;+\frac{1}{2}\sum_{i\in N}\gamma_{i}\sum_{j\in N_{i}}\alpha_{ji}(r_{j}^{T}r_{j}\\ \;+{\dot{\tau }}_{ji}(t)r_{j}^{T}(t-\tau_{ji}(t))r_{j}(t-\tau_{ji}(t)))\\ \;\leq -\frac{1}{2}\sum_{i\in N}\gamma_{i}\sum_{j\in N_{i}}\alpha_{ji}{\left(.\right)}^{T}\left({\dot{\in }}_{ij}+\lambda \epsilon_{ij}\right)\\ \;+\frac{1}{2}\sum_{i\in N}\gamma_{i}\sum_{j\in N_{i}}\alpha_{ji}\left(-r_{i}^{T}r_{i}+r_{j}^{T}r_{j}\right)\\ \;+\frac{1}{2}\sum_{i\in N}\gamma_{i}\sum_{j\in N_{i}}\alpha_{ji}\left(\psi \;r_{j}^{T}(t-\tau_{ji}(t))r_{j}(t-\tau_{ji}(t))\right) \end{array}$$

knowing that based on *assumption 4*, the upper-bound of ${\dot{\tau }}_{ji}\left(t\right)$ is ψ.

Now, by adding and subtracting the term,

$$\frac{1}{2}\underset{i\in N}{\sum }\gamma_{i}\underset{j\in N_{i}}{\sum }\alpha_{ji}\psi \left(r_{i}^{T}\left(t-\tau_{ii}\left(t\right)\right)\times r_{i}\left(t-\tau_{ii}\left(t\right)\right)\right)$$

in inequality (43), the following inequality is obtained

(44) $$\begin{array}{l} \sum_{i\in N}r_{i}^{T}{\bar{\tau }}_{c_{i}}\\ \;+\frac{1}{2}\sum_{i\in N}\gamma_{i}\sum_{j\in N_{i}}\alpha_{ji}(r_{j}^{T}r_{j}-r_{j}^{T}(t-\tau_{ji}(t))r_{j}(t-\tau_{ji}(t))\\ \;+{\dot{\tau }}_{ji}(t)r_{j}^{T}(t-\tau_{ji}(t))r_{j}(t-\tau_{ji}(t)))\\ \;\leq -\frac{1}{2}\sum_{i\in N}\gamma_{i}\sum_{j\in N_{i}}\alpha_{ji}{\left(.\right)}^{T}\left({\dot{\epsilon }}_{ij}+\lambda \epsilon_{ij}\right)\\ \;+\frac{1}{2}\sum_{i\in N}\gamma_{i}\sum_{j\in N_{i}}\alpha_{ji}\left(-r_{i}^{T}r_{i}+r_{j}^{T}r_{j}\right)\\ \;+\frac{1}{2}\sum_{i\in N}\gamma_{i}\sum_{j\in N_{i}}\alpha_{ji}\psi (\;r_{j}^{T}(t-\tau_{ji}(t))r_{j}(t-\tau_{ii}(t))\\ \;-r_{i}^{T}(t-\tau_{ii}(t))r_{i}(t-\tau_{ji}(t)))\\ \;+\frac{1}{2}\sum_{i\in N}\gamma_{i}\sum_{j\in N_{i}}\alpha_{ji}\psi \left(\;r_{i}^{T}(t-\tau_{ii}(t))r_{i}(t-\tau_{ii}(t))\right) \end{array}$$

Three notes are to be considered. First, the self delays of operators are negligible, i.e., τ_*ii*_ ≃ 0. The second factor is that using *Remark 2*, $\frac{1}{2}\underset{i\in N}{\sum }\gamma_{i}\underset{j\in N_{i}}{\sum }\alpha_{ji}\left(f_{j}-f_{i}\right)=\frac{1}{2}\gamma_{b}^{T}\;L\;f$ and is equal to zero.

Substituting (44) in (42) and using the constraint (36), the Lyapunov derivative becomes:

$$\begin{array}{c} \dot{V}\leq -\frac{1}{2}\underset{i\in N}{\sum }\underset{j\in N_{i}}{\sum }\gamma_{i}\alpha_{ji}\epsilon_{ij}^{T}\epsilon_{ij}-\frac{1}{2}\underset{i\in N}{\sum }\underset{j\in N_{i}}{\sum }\gamma_{i}\alpha_{ji}{\dot{\epsilon }}_{ij}^{T}{\dot{\epsilon }}_{ij}\\ -\underset{i\in N}{\sum }r_{i}^{T}\left(k_{i}-\psi \gamma_{i}\underset{j\in N}{\sum }\alpha_{ji}\right)r_{i}\leq 0 \end{array}$$

Therefore, based on Lyapunov theory, *r*_*i*_(*t*) asymptotically converge to zero, which completes the proof.

    □

## 6. Novel Design for Simultaneous Training and Therapy in Telerehabilitation Tasks

The main idea that led to the concept of “Simultaneous Training and Therapy,” came to the minds of the authors of this article after several attending the clinics and closely observing the trainees and the rehabilitating patients in the field. The main problem was the presence of a large number of trainees and their short training time. Therefore, the use of manipulators in the TR process for trainees, patients, and therapists can significantly reduce the cost of patients attending the clinic and the cost of one-to-one teaching for trainees as well as its duration time.

Consequently, this is the most important section, and in fact the practical conclusion of this article, because it implements the main idea of the authors. To show the effectiveness of the proposed method in this article, various examples in the field of rehabilitation will be given along with practical experiments.

Therefore, to show the effectiveness of the proposed method in the sections 4 and 5, utilizing the power of theoretical parts achieved, some novel designs in the simultaneous training and therapy for TR systems are proposed. Two tuning matrices *L* and *D* as *Laplacian* and *Sensed Force*, are used to implement such schemes. For *Laplacian* matrix *L*, it is enough to be connected, as mentioned in *Remark 2*. The tuning matrix *D* has a decisive role in the TR scenarios.

Based on the controllers in *Theorems 1–3*, we have the freedom to design multiple scenarios for the TR tasks. The primary item in this structure that gives the freedom, is the matrix *D*, which can be used in designing the remote rehabilitation structure. It has been shown that the controller guarantees the position synchronization. As described in *Remark 5*, by selecting a suitable matrix *D*, we can design the desired *Sensed Forces* at a steady-state as the following:

$$F_{des}\left(\infty \right)=\left[L^{T}PL\right]Q\left(\infty \right)=D.Q\left(\infty \right)$$

the desired force is achieved, which is a function of operator position errors. Thus, the equation *D* = *L*^*T*^*PL* should be solved by choosing a proper positive (semi-)definite matrix *P*. However, it is already known from *Remark 1*, the Laplacian matrix is singular by its nature. Therefore, the following remark is to be noted.

**Figure 4:**
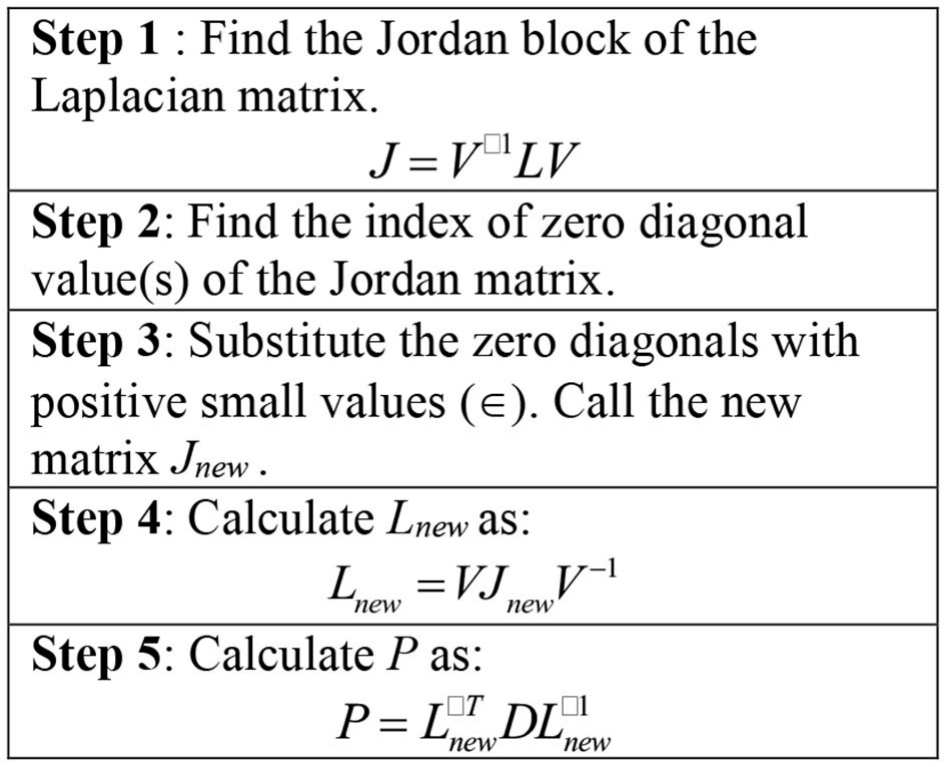
The steps to calculate the Positive semi-definite matrix *P*.

Remark 8. *Applying the Theorems 1–3, to ensure the stability of the system, the matrix *P* should be positive semi-definite. As stated in *Remark 1*, all of eigen-values associated to *L* are positive or zero. So, *L* is a positive semi-definite (Golub and Van Loan, 1996). Adding a small positive value to zero eigenvalue(s) of *L* retains the Laplacian matrix being positive definite. In addition, the desired force matrix (*D*) is chosen as a positive definite matrix. Therefore*, $P=L_{new}^{-T}DL_{new}^{-1}$ *would be positive semi-definite. The algorithm is depicted in Figure 4*.

(45) $$\begin{array}{l} \tau_{11}\left(t\right)=0.2\left(1+\sin\left(0.2t\right)\right)s,\;\tau_{12}\left(t\right)=0.6\left(0.75+.5\sin\left(t\right)\right)s,\;\tau_{13}\left(t\right)=0.5\left(0.3+.05\sin\left(0.6t\right)\right)s\\ \tau_{21}\left(t\right)=0.3\left(1+0.25\sin\left(0.2t\right)\right)s,\;\tau_{22}\left(t\right)=0.1\left(1+0.3\sin\left(0.5t\right)\right)s,\;\tau_{23}\left(t\right)=0.12\left(1+0.4\sin\left(0.6t\right)\right)s\\ \tau_{31}\left(t\right)=0.27\left(1+0.28\cos\left(t\right)\right)s,\;\tau_{32}\left(t\right)=0.23\left(0.5+0.1\sin\left(0.3t\right)\right)s,\;\tau_{33}\left(t\right)=0.5\left(0.11+0.01\sin\left(0.7t\right)\right)s \end{array}$$

Up to now, the centralized and decentralized controllers were proposed that can accommodate various multi-lateral TR for several users, including patient, trainees, and therapist interaction using a multi-DOF tele-robotic system. The authority sharing structure in related papers like in Khademian and Hashtrudi-Zaad (2012), Li et al. (2015), and Hashemzadeh et al. (2016) can be regarded as a particular case of the current research by applying matrices *D, L*, and *P* ≥ 0. For example, the one proposed in Hashemzadeh et al. (2016) can be implemented in the structure of this paper by considering

(46) $$L=\;\left[\begin{array}{ccc} 1 & \alpha -1 & -\alpha \\ -\alpha & 1 & \alpha -1\\ \alpha & \alpha -1 & 1 \end{array}\right]$$

The above equation directly points to Equation (5) of Hashemzadeh et al. (2016); so, the remark 2 is satisfied. To achieve Equations (6)–(8) of the mentioned paper, it is easy to consider *D* as follows

$$D=\left[\begin{array}{ccc} 1 & -\alpha & 0\\ -(1-\alpha ) & 1 & 0\\ -\alpha & -(1-\alpha ) & 0 \end{array}\right]L$$

Therefore, considering the algorithm in Figure 4, the matrix *P* will be calculated as follows:

$$P=\left[\begin{array}{ccc} 1 & 0 & 0\\ 0 & 1 & 0\\ 0 & 0 & 0 \end{array}\right]$$

It is obvious that the matrix *P* is positive semi-definite. So, it can be used in the Lyapunov function (26). Furthermore, by considering exactly the same *L* as in (46), for Equations (10)–(12) of Khademian and Hashtrudi-Zaad (2012) and considering the following *D* for Equations (13)–(15) of the mentioned paper, the system can be implemented easily.

$$D=\left[\begin{array}{ccc} 1 & 1-\alpha & \alpha \\ \alpha -1 & 1 & -\alpha \\ -\alpha & \alpha -1 & 1 \end{array}\right]$$

Thus, considering the algorithm in Figure 4, the matrix *P* will be calculated as follows:

$$P=\left[\begin{array}{ccc} \frac{1}{\alpha^{2}-\alpha +1} & \frac{-2\alpha^{2}+\alpha +2}{2\alpha \left(\alpha^{2}-\alpha +1\right)} & \frac{-(\alpha -2)}{2\alpha \left(\alpha^{2}-\alpha +1\right)}\\ \frac{\alpha }{\alpha^{2}-\alpha +1} & \frac{\alpha^{2}+1}{2\alpha^{2}\left(\alpha^{2}-\alpha +1\right)} & \frac{-(\alpha^{2}-1)}{2\alpha^{2}\left(\alpha^{2}-\alpha +1\right)}\\ 0 & \frac{1}{2\alpha^{2}} & \frac{1}{2\alpha^{2}} \end{array}\right]$$

**Figure 5:**
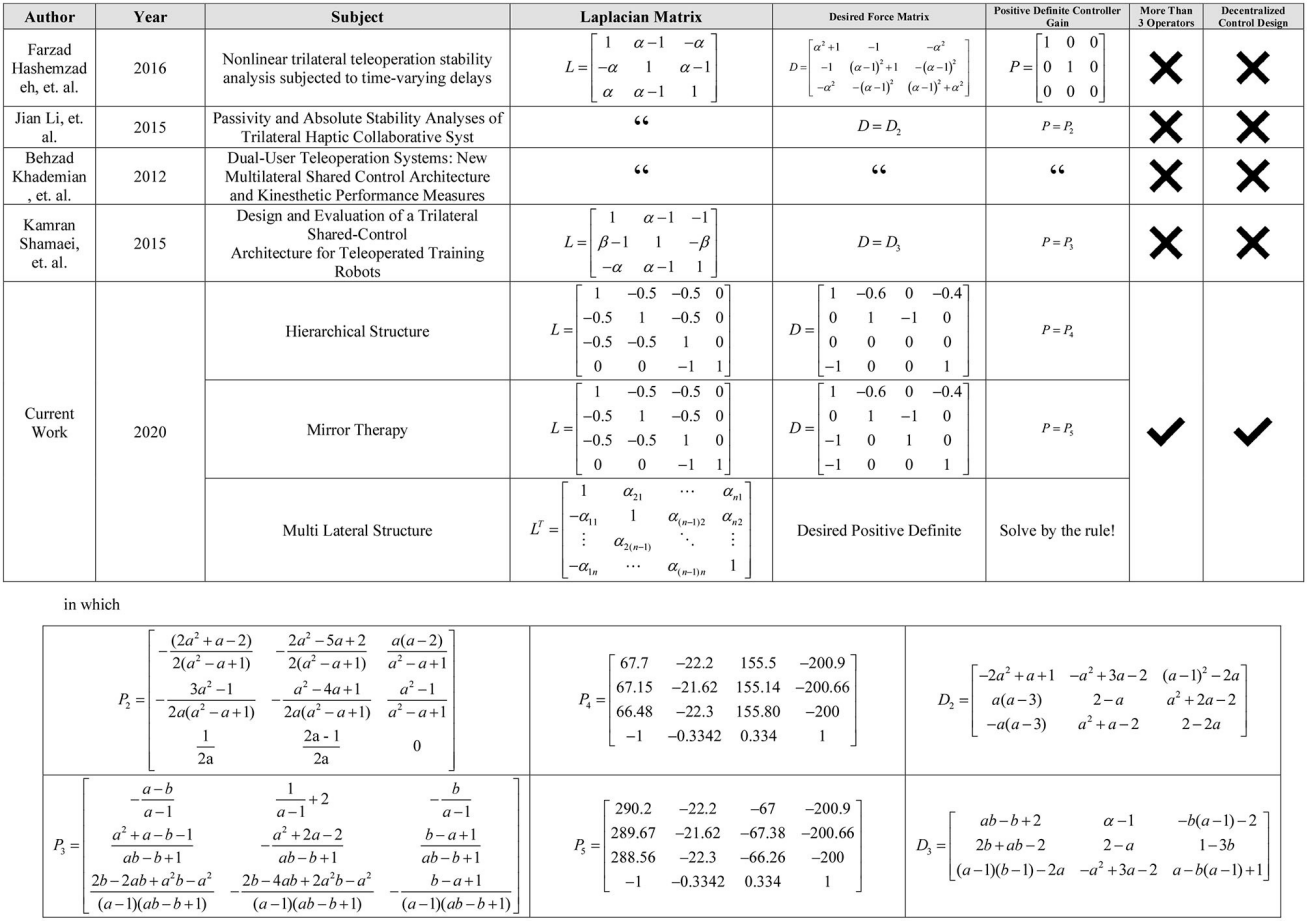
Table of comparison of similar works.

It is easy to verify that the leading principal minors of *P* are all positive, guaranteeing that the matrix *P* is positive definite in this example. More comparisons with similar existing frameworks are illustrated in Figure 5 at the end of the paper.

In addition, to implement the structure of the proposed method, the *shared environment* is used for all the experiments in this section. To implement the shared environment, the model of virtual manipulator, and impedance of the environment, a software called Unity3D^©^ is used. Furthermore, the controller is implemented in Simulink Desktop Real-Time^TM^ (Sahin et al., 2017), and it is connected to Unity3D^©^ via the UDP protocol. The delays considered in the system for all of the experimentation are as in (45), which obviously satisfies *Assumption 4*. The participants in all of the experimentation are healthy people emulating the behavior of the therapist, patient, and trainees inside the virtual environment^1^. The proposed structure will be examined in the succeeding subsections for some novel rehabilitation scenarios.

### 6.1. Design and Control of Hierarchical Telerehabilitation Systems

**Figure 6:**
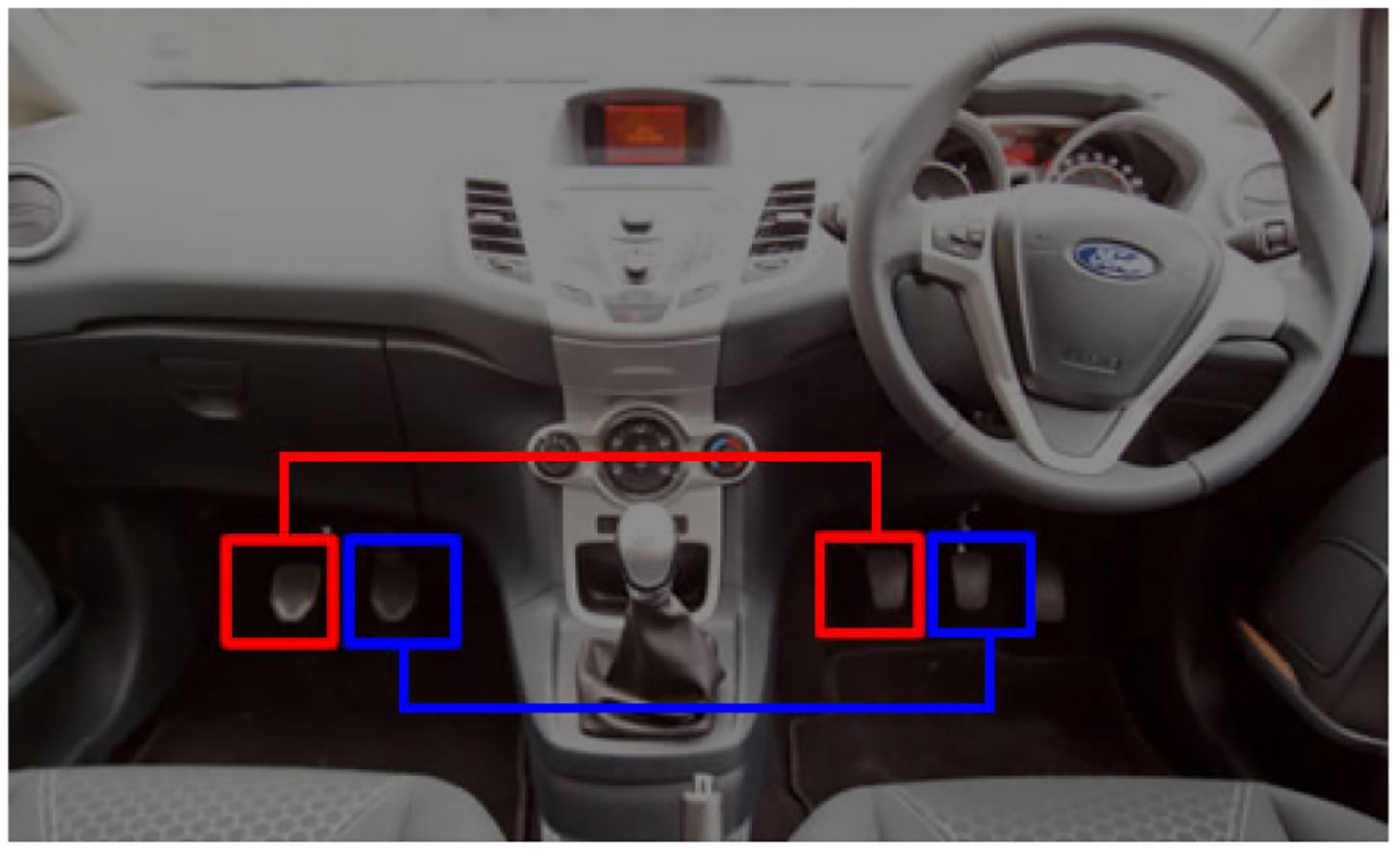
There are pedals under the feet of the trainee and the instructor in driving school. However, if the instructor wishes, he/she can depress each pedal even though the instructor has not depressed it. So, there exist a hierarchy between the instructor and the trainee.

The idea of the Hierarchical Telerehabilitation System (HTS) is similar to the idea of driving instruction in driving school. In the training cars, a dual pedal is placed under the instructor's feet, and the instructor can override the trainee's pedals, meaning that a hierarchy exists between the instructor and the trainee (Figure 6). The trainee cannot affect the pedal of the instructor, while the instructor can depress his/her pedal and override the trainee's pedal. This idea has been used for the HTS. However, in the HTS, three users participate in the process instead of two users, i.e., therapist, trainee, and patient. In this hierarchy, the therapist has the highest rank, and the patient has the lowest rank. So, the therapist can override the movements of the trainee and the patient. And, the trainee can override the movements of the patient.

On the other hand, the virtual environment interacts with the patient and put him/her in a predetermined path. So, the virtual environment can play a decisive role in this process. Many conventional rehabilitation therapies can be implemented using the HTS. Two of them are “teach and repeat therapy” and “assist as need therapy.” For teach and repeat therapy, the virtual environment can be trained by an expert therapist's hand movements (record the movement task) in periodic tasks, e.g., moving on a circle or square. After the therapist leaves the process, the virtual environment repeats the therapist's hand movements. The virtual environment can also play the role of “assist as need therapy” (Staubli et al., 2009). It means that if the patient's movement is in the desired path, no extra force is exerted to the patient's hand. However, if the patient's movement error exceeds a specified limit, the virtual environment assists the patient's hand return to the desired path. This can be implemented easily by choosing the appropriate functions for matrix *D*.

**Figure 7:**
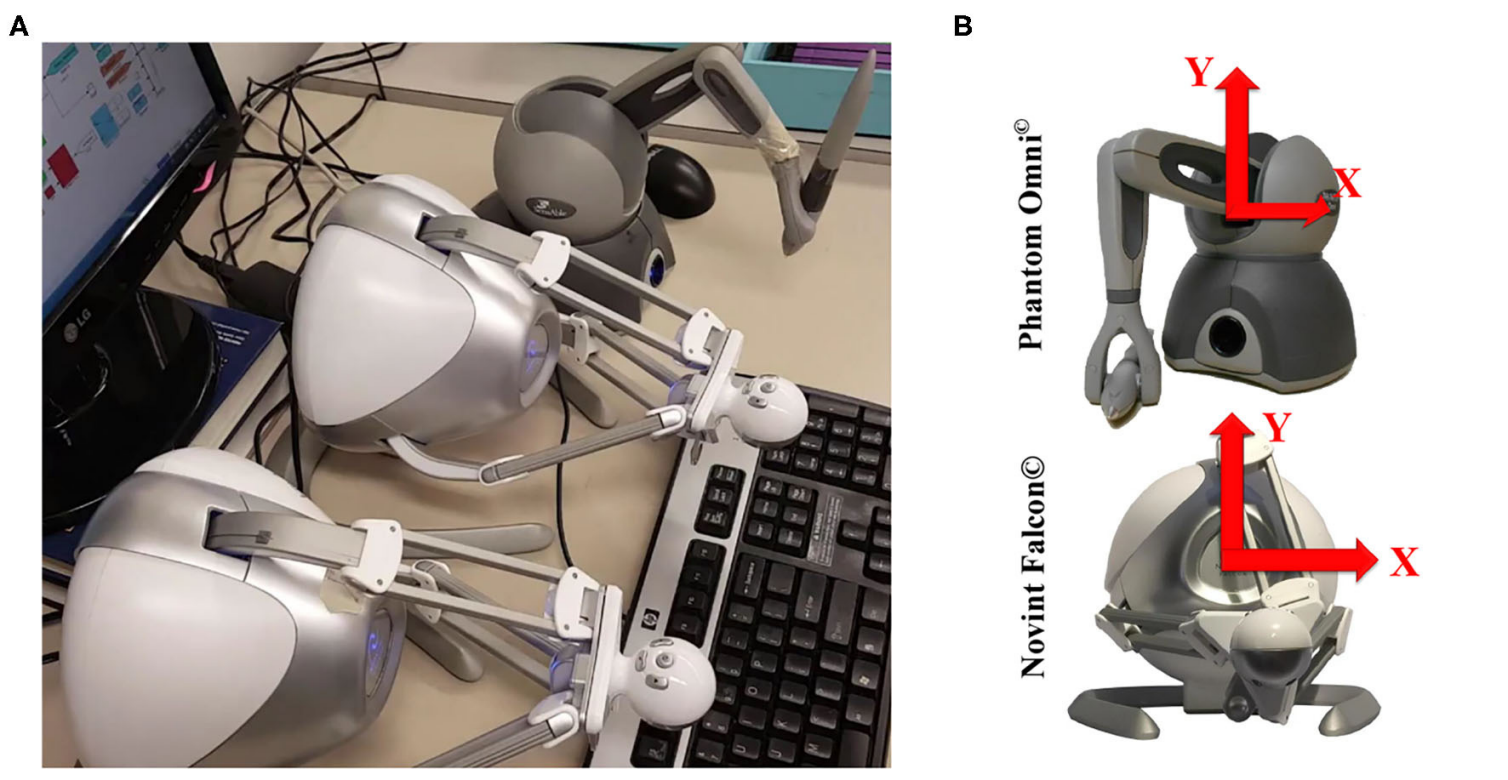
Implementation of the HTS. In part **(A)**, the overview of three non-homogeneous robots, two of which are Novint Falcon^©^ and one Phantom^©^, is shown. The *X* and *Y* coordinate frames, which represent two-dimensional motion, are assigned to them in part **(B)**.

To show the performance of the HTS, a practical scenario is proposed. Three operators consisting of a therapist (operator 3), a student/trainee (operator 2), and a patient (operator 1) are considered. These operators are working in a shared virtual environment (operator 4). In this experiment, operator 1 has the highest rank while the operator 4 has the lowest rank. Additionally, the robots considered for these experiments are non-homogeneous, including one Phantom Omni® and two Novint Falcons®, interacting with the Therapist, Trainee, and the Patient, respectively (Figure 7). The experimental parts are described in the Appendix. The desired matrix of the sensed force and the Laplacian matrix are selected for position synchronization as follows

(47) $$\begin{array}{cc} L=\left[\begin{array}{cccc} 1 & -0.5 & -0.5 & 0\\ -0.5 & 1 & -0.5 & 0\\ -0.5 & -0.5 & 1 & 0\\ 0 & 0 & -1 & 1 \end{array}\right] & D=\left[\begin{array}{cccc} 1 & -0.6 & 0 & -0.4\\ 0 & 1 & -1 & 0\\ 0 & 0 & 0 & 0\\ -1 & 0 & 0 & 1 \end{array}\right] \end{array}$$

**Figure 8:**
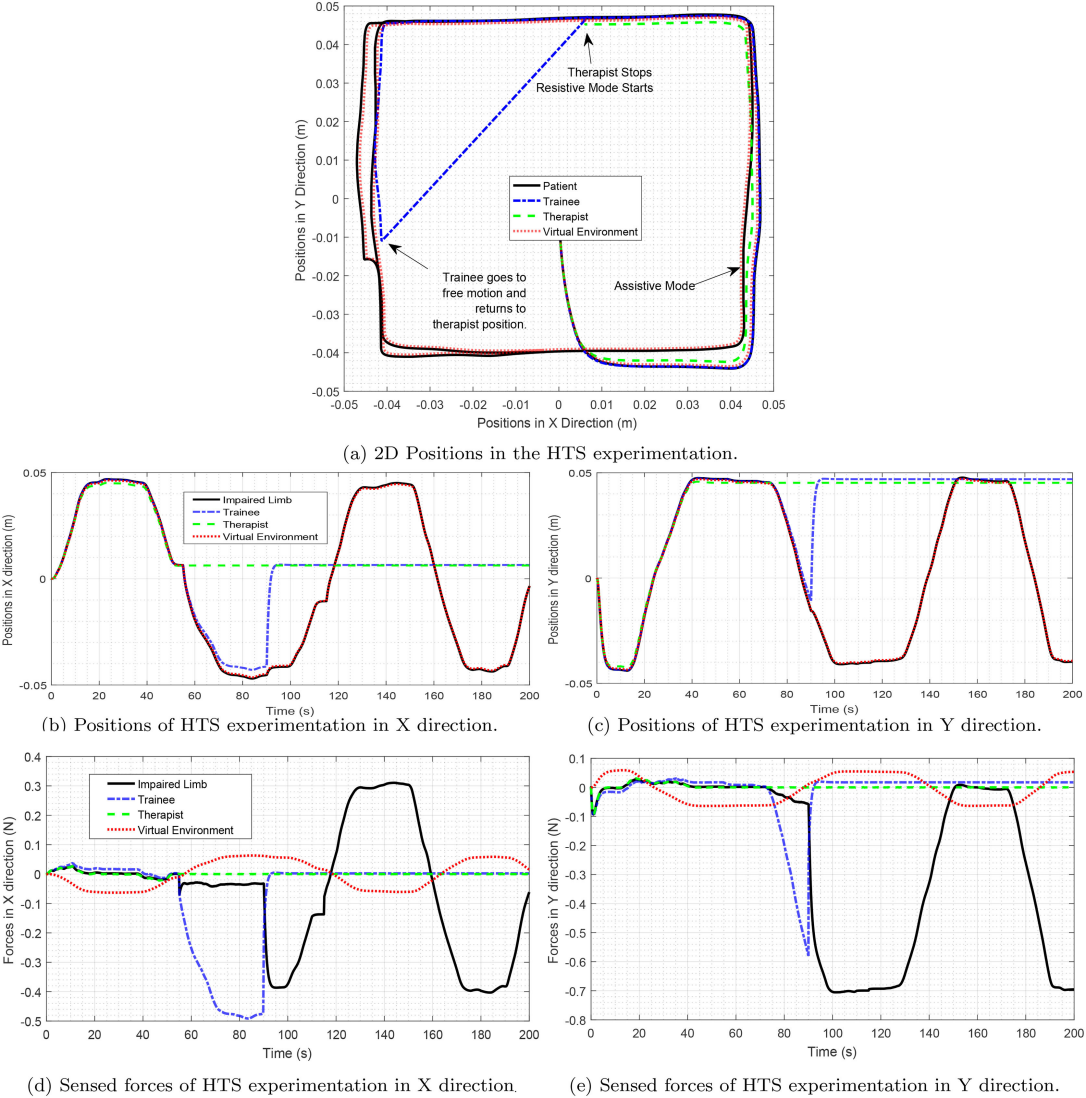
Forces in hierarchical therapy. This figure illustrates three phases of therapy. In the first phase, all the operators participate in the TR process. So, the therapist assists all of them to move in the correct path. In the second phase, when the therapist stops moving, the trainee's force is of larger magnitude. Moreover, in the third phase, the patient's force increases. The phase stage, is resistive for the trainee, that helps them to learn the process of rehabilitation. The third phase, is resistive for the patient trainee.

By looking at Laplacian matrix *L* it is easy to verify that the *Remark 2* is satisfied. The third row of matrix *D* is totally zero, showing that the therapist's desired sensed force is not affected by other operators. The results of the experiments are shown in Figure 8. As depicted in the figures in the first phase, the positions of both the trainee and the patient follow the position of the therapist, and the system assists both of them for moving. In the second phase, the therapist stops moving and the trainee goes to the resistive phase, while the patient is still in the assistive mode. So, the trainee should enforce a larger amount of effort to move in the direction. In the third phase, both the therapist and the trainee stop moving, and the patient is asked to move. Therefore, the patient goes to the resistive mode and the amount of the patient's force becomes larger. So, both assistive and resistive scenarios can be implemented in this method.

### 6.2. Teach and Repeat Therapies

**Figure 9:**
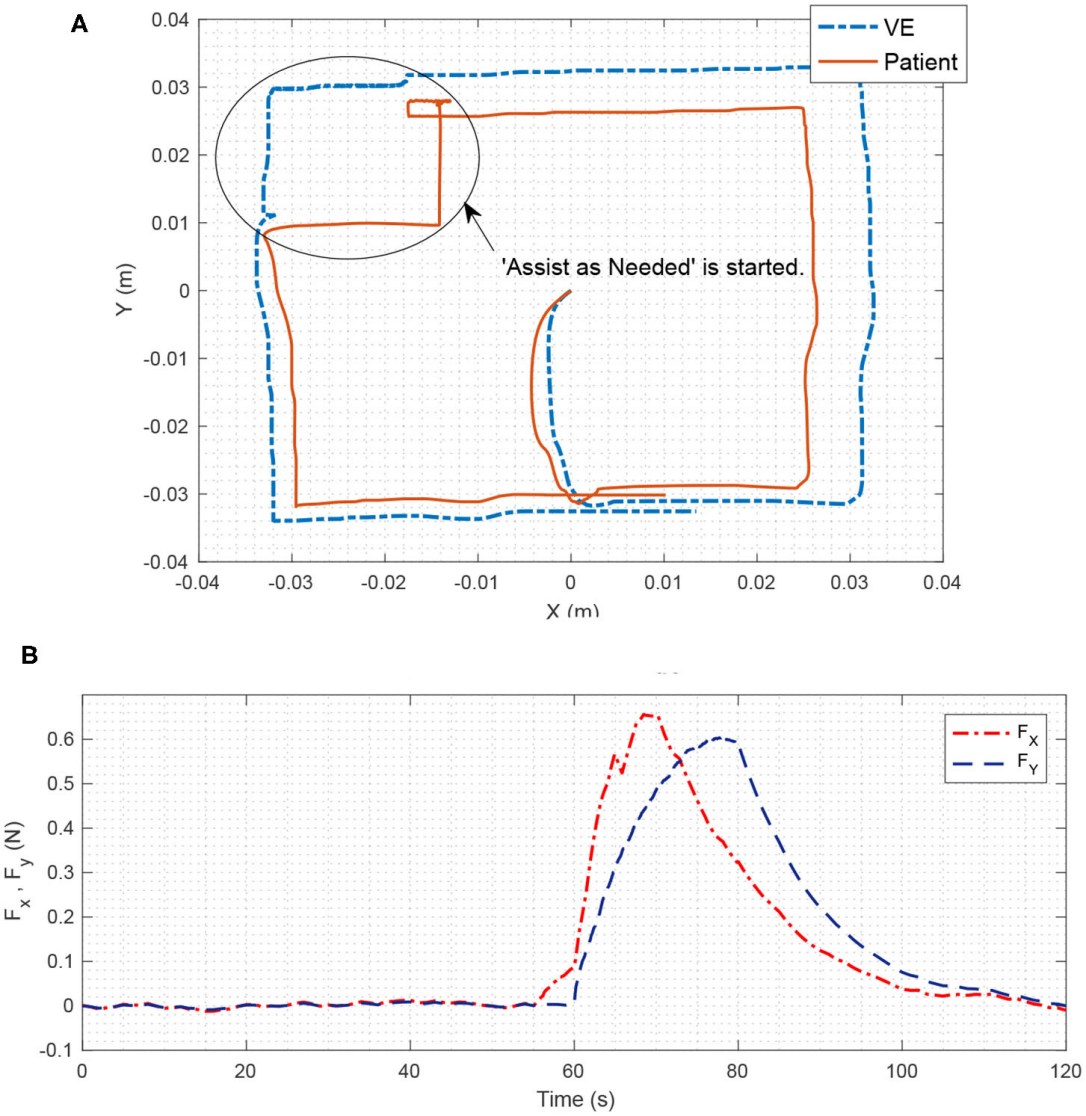
**(A)** The 2D position of “Assist as Needed” is shown. **(B)** Assistive force in X and Y direction is illustrated. In the first 60 s of the therapy, teach and repeat method is applied. The path is recorded in the VE and is replied to the patient. The patient moves freely in the specified path, which is a square here. The VE also moves on the square. If the patient's movement error is greater than the specified limit, Assist as Needed force is activated and attempts to return the patient's robot position to the original square path with assistive force. As can be seen in part **(B)** the assistive force is almost zero before the 60th second; however, from about 60th second, it is activated in the X and Y directions and tries to return the patient to the desired path. Note that, in this figure, the absolute values of the assistive forces are shown to the reader for better understanding.

The virtual environment proposed in this project has the ability to store the therapist's hand movements and then replay it for the rehabilitation process (Babaiasl et al., 2016). Therefore, the virtual environment can play the role of “teach and repeat.” In the experiment performed as the teach and repeat role, a square path of the therapist's hand movements in section 6.1 is stored and then replayed in the rehabilitation process. Moreover, as can be seen from Figure 9, the teach and repeat therapy, was performed in the first 60 s of this experiment. Due to the capability of this method, there would be freedom for the therapist to put the process in teach and repeat mode and observe the process without his/her intervention.

### 6.3. Assist as Needed

During the replay discussed in the experiment of section 6.2, the patient follows a square path, and if he/she deviates from the specified path, the assistive force returns the patient's hand to the square path, which is “Assist as Needed” therapy (Luo et al., 2019). To implement such therapy with our proposed method, consider a case study with similar participants as section 6.1. Then, the following switching criteria for matrix *D* is chosen.

(48) $$D=\{\begin{array}{cc} 0_{4\times 4} & \text{if }e\leq \rho \\ \left[\begin{array}{cccc} 1 & 0 & 0 & -1\\ 0 & 0 & 0 & 0\\ 0 & 0 & 0 & 0\\ 0 & 0 & 0 & 0 \end{array}\right] & \text{if }e>\rho \end{array}$$

If the tracking error (*e*) is less than the allowable limit (ρ), the matrix *D* is set to **0**_4×4_. Conversely, if the tracking error is greater than the specified limit, the first line of matrix *D*, which is related to the patient's hand, would changes as [$\begin{array}{cccc} 1 & 0 & 0 & -1 \end{array}$], meaning that the virtual environment tries to return it to the main path. The other zero rows, mean that other operators move freely without getting any force feedback.

As can be seen in Figure 9, in the 60th second, the patient is out of the marked square path (*e* > ρ), and the assistive force returns the patient's hand to the main path. When the patient returns to the square path, the assistive force will gradually vanish from the rehabilitation process.

### 6.4. Supervised Mirror Therapy

In this part, the scenario of Supervised Mirror Therapy (SMT) is implemented. In SMT, the patient attempts bi-manual symmetric movements as moving in the mirror trajectory. Meanwhile, the (remote) therapist helps the patient to move his hand in a desired trajectory. The manipulators keep the limbs in symmetry that helps the affected limb to rehabilitate. For the sake of synchronization in this SMT, the desired sensed force matrix *D* and the Laplacian matrix *L* are selected as follows:

(49) $$\begin{array}{cc} L=\left[\begin{array}{cccc} 1 & -0.5 & -0.5 & 0\\ -0.5 & 1 & -0.5 & 0\\ -0.5 & -0.5 & 1 & 0\\ 0 & 0 & -1 & 1 \end{array}\right] & D=\left[\begin{array}{cccc} 1 & -0.6 & 0 & -0.4\\ 0 & 1 & -1 & 0\\ -1 & 0 & 1 & 0\\ -1 & 0 & 0 & 1 \end{array}\right] \end{array}$$

**Figure 10:**
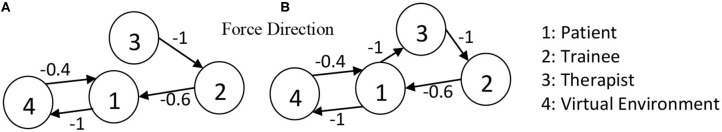
Desired graphs, considered for the proposed system in sections 6.1 and 6.4. This diagram is equivalent to the *D* matrices in (47) and (49) in which, the circles represent the role of each user. Next to the diagram, meaning of the numbers inside each circle is written. Moreover, on the arrows in the diagram, numbers are written that are equal to the numbers expressed in the rows of the D matrices of (47) and (49). Part **(A)** shows the force graph of HTS. It is the graph of matrix *D* in (47). Part **(B)** depicts the force graph of the proposed SMT. It is the graph of matrix *D* in (49).

By looking at Laplacian matrix *L* it is easy to verify that the *Remark 2* is satisfied. The only difference between (47) and (49) is the third row of the matrix *D*, meaning that the therapist's sensed force is a function of the patient's position (see Figure 10). So, the concept of unilateral teleoperation is changed to multi-lateral teleoperation, because the desired force forms a closed-loop structure. The varying delays in the channels are considered as (45), and remaining delays in the channels are selected as (50).

(50) $$\begin{array}{l} \tau_{14}(t)=0.5(0.3+\;.05\text{ sin}(0.6t))s,\;\tau_{24}(t)=0.5(0.11+0.01\text{ sin}(0.7t))s,\;\tau_{34}(t)=0.23(0.5+0.1\text{ sin}(0.3t))s\\ \tau_{41}(t)=0.3(1+0.25\text{sin}(0.2t))s,\;\tau_{42}(t)=0.12(1+0.4\text{sin}(0.6t))s,\;\tau_{43}(t)=0.23(0.5+0.1\text{ sin}(0.3t))s\\ \tau_{31}(t)=0.5(0.11+0.01\text{sin}(0.7t))s \end{array}$$

**Figure 11:**
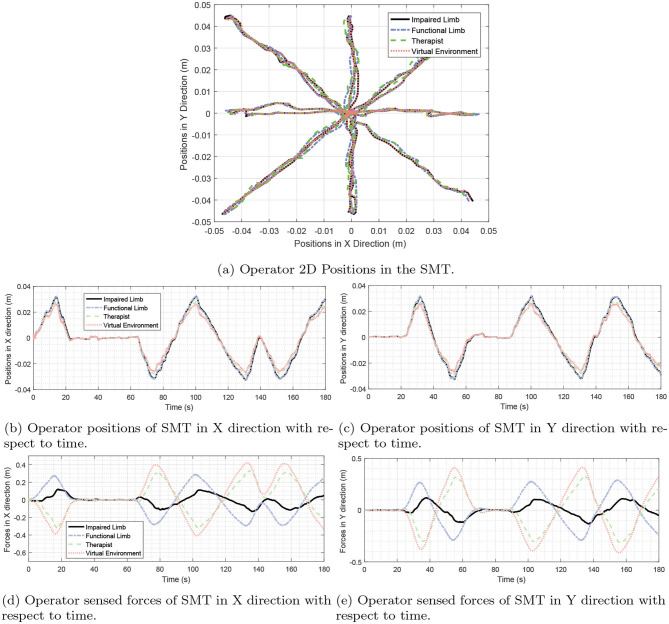
SMT experimentation. Participants in this TR process include a therapist, a functional hand, and impaired hand. Also, the virtual environment as the last operator in this process cooperates with other operators. As shown in the figure, there is a star path for users to move. Part **(a)** Shows the movement of the operators in two dimensions and part **(b,c)** show the movement of the operators in the X and Y dimensions separately. Sections **(d,e)** also show the force of the operators in the x and y directions. As can be seen, according to Equation (51), the sum of the forces in each case will be close to zero.

The results of the experiments and the 2D plots of positions of the operators are depicted in Figure 11. It is demonstrated that the hands of the patient are aligned with the positions of the hand of the therapist. At the steady-state, the operator forces are such that the summation of the forces will be zero.

(51) $$\begin{array}{l} \tau_{\text{Therapist}}+\tau_{\text{ImpairedLimb}}+\tau_{\text{FunctionalLimb}}+\tau_{\text{SVE}}=0 \end{array}$$

The above equation is easily verifiable through (15) and (49). This is reflected in Figures 11D,E.

### 6.5. Several Trainees in Telerehabilitation Process

**Figure 12:**
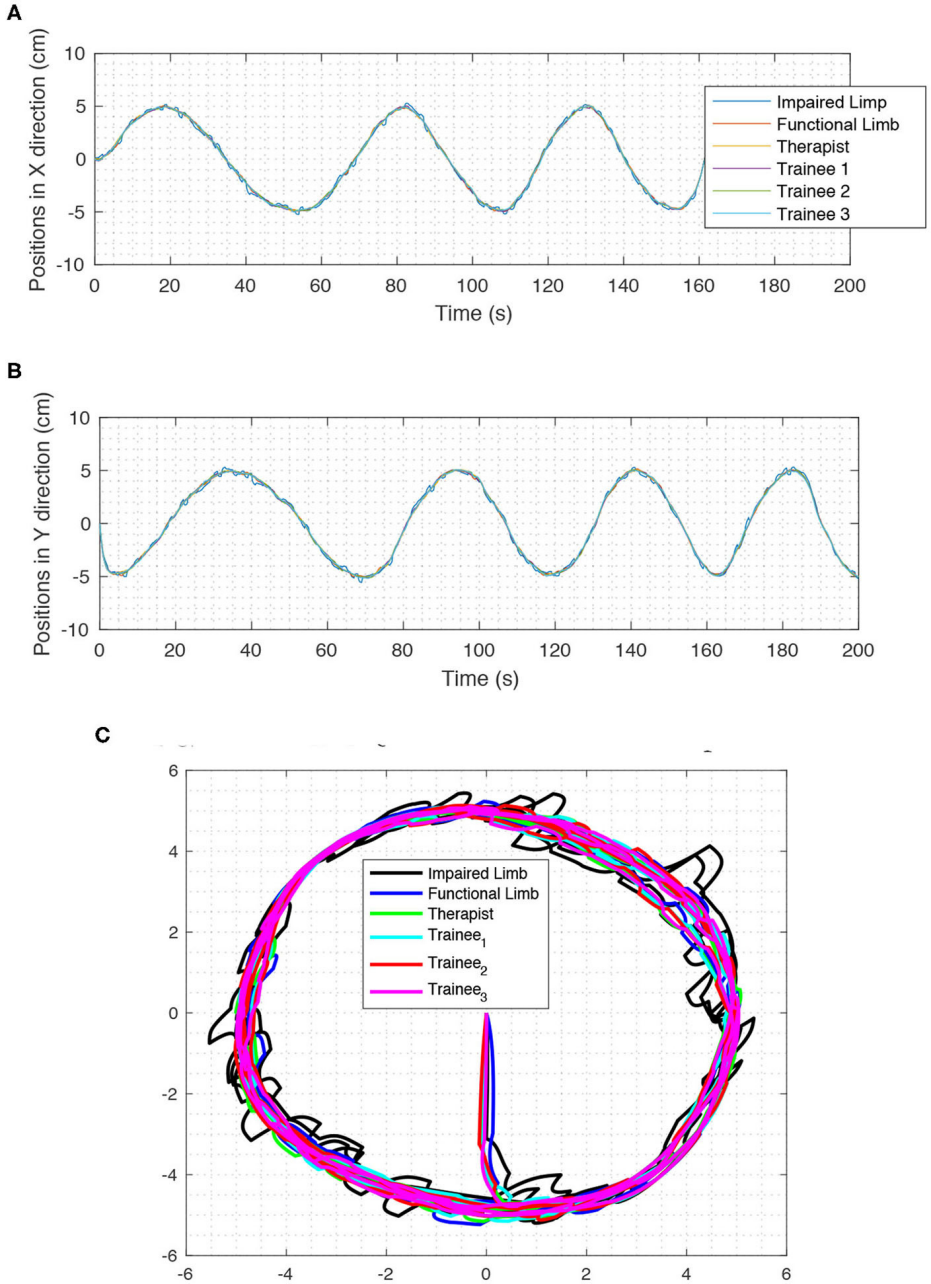
The positions in STTRP. In this process, six operators participate in TR, simultaneously. impaired hand, functional hand, Therapist, and trainee #1, participate in the force interaction of TR process, and neither of these four operators is superior to the other. Operator #2 and #3 do not participate in the force interaction. **(A)** Positions in X direction in STTRP experiment. **(B)** Positions in Y direction in STTRP experiment. **(C)** Positions in 2D space in STTRP experiment (XY Direction).

In this part, the scenario called *Several Trainees in Telerehabilitation Process* (STTRP) is introduced. The idea of STTRP is based on the fact that, while the patient is undergoing the process of stroke recovery, several numbers of the trainees can learn the required skills via robots without interrupting the interaction of the patient and the expert therapist. The proposed system forces the trainee's position to track the desired position and sense the desired force of the system. The numbers of trainees may vary from 0 to any number. By choosing the correct matrix *D*, the trainees sense exactly what the expert therapist wants to teach them without interfering in the rehabilitation process. By advancing the process of therapy, one or more trainees can participate more efficiently in the process. The scenario for this experiment is tracking a circular path in 2-D space. All the operators move in the same direction, and the positions are almost a circle. The experimental results are depicted in Figure 12 which shows the impaired limb (black route) will finally move neatly on the circular path after some iterations. So, the experiment confirms the stability and synchronization of operators.

## 7. Conclusion

In this paper, the problem of multi-lateral TR with non-linear and uncertain dynamics was addressed. To deal with the theoretical parts of such systems, a novel structure based on the MAS was presented. This structure could solve the complexity of multi-lateral rehabilitation system due to several numbers of operators in the process. The key factor in the MAS is the *self-intelligence* between the agents that shows the consciousness of each agent about the other ones. Moreover, uncertainties in the operators' dynamics, as well as time-varying delays in the communication channels, were addressed by using the power of the MAS and passivity based adaptive controls. Furthermore, this paper introduced a framework for simultaneous training and therapy in multi-lateral TR systems. The method can be used in medical education centers. It could help the trainee to be involved in a “hands-on” manner during the rehabilitation process by an expert therapist. So, they were introduced and tested particularly with the tuning parameters *L* (Laplacian Matrix) and *D* (Sensed Force Matrix) that verified the reliability and performance of the proposed framework. All the experimentation were accomplished with the volunteer students in the “Telerobotic and Biorobotic Systems Lab.” Because of acceptable results, in the near future, the experimentation will be implemented in clinical centers and on real patients.

## Data Availability Statement

The raw data supporting the conclusions of this article will be made available by the authors, without undue reservation, to any qualified researcher.

## Ethics Statement

The studies involving human participants were reviewed and approved by All subjects provided informed consent to the experimental procedures, which were reviewed and approved by the University of Alberta Research Ethics Board (Study ID: Pro00033955). The patients/participants provided their written informed consent to participate in this study.

## Author Contributions

IS conceived of the presented idea and developed the theory and performed the computations. HT and MT verified the analytical methods. RP encouraged HT and MT to investigate experimental scenarios and supervised the findings of this work. IS, HT, and MT carried out the experiment. IS wrote the manuscript with support from MT, HT, and RP. All authors discussed the results and contributed to the final manuscript.

## Conflict of Interest

The authors declare that the research was conducted in the absence of any commercial or financial relationships that could be construed as a potential conflict of interest.

## Figures and Tables

**Figure A1:**
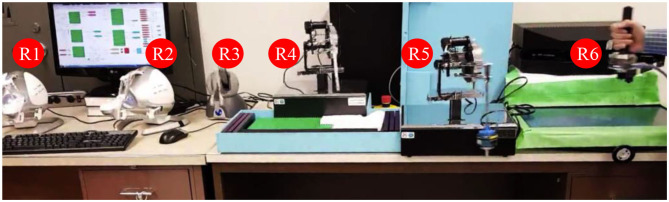
In this figure, six non-homogeneous robots participate in a rehabilitation process as discussed in Figure A2. A dual set of Novint Falcon®, one Phantom Omni®, Two Phantom Premium® robots, and one Quanser® robot participate in the process, forming R1–R6, respectively.

**Figure A2:**
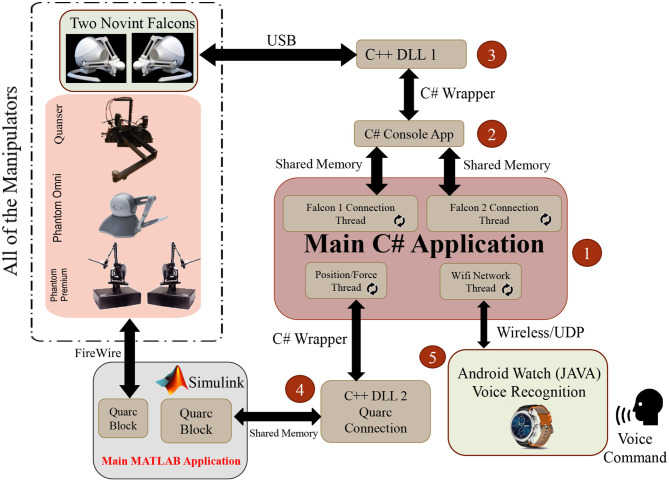
Overview of the implemented software for the proposed method. As it is seen, the manipulators are selected in a non-homogeneous manner. That is, a dual set of Novint Falcon®, one Phantom Omni®, Two Phantom Premium® robots, and one Quanser® robot are provided for this purpose (R1–R6 in Figure A1). The software written for this system consists of five parts, all of which have been prepared and implemented by the authors of this article. Bilateral arrows represent the two-way communication between the marked blocks. For example, Novint Falcon robots send/receive data to/from the main system through the USB protocol and via the intermediate programs “C++ DLL 1” (Block #3) and “C# Console App” (Block #2). Block #3 and Block #2 are connected to each other via C# wrapper, and Block #2 is connected to the Block #1 via the shared memory. All the other blocks are connected to the robots in similar situation as shown in this figure.

## Footnotes

^1^ All subjects provided informed consent to the experimental procedures, which were reviewed and approved by the University of Alberta Research Ethics Board (Study ID: Pro00033955).

## 8. Appendix: Experimentation Setup

All the experiments performed in this article have been implemented by the hardware and software described in this subsection. The experiments were implemented with capability of handling six non-homogeneous manipulators consisting of a dual set of Novint Falcon® (Karbasizadeh et al., 2018), one Phantom Omni®, Two Phantom Premium® robots (Taati et al., 2008), and one Quanser® robot (Atashzar et al., 2017), respectively (Figure A1). A software was developed in Windows platform that can connect and control the hardwares used in the experiments. The overview of the software is given in Figure A2. It is composed of five interactive modules. The main application (Block #1) is written in C# language and is based on multi-threaded programming. It is connected to C# console application (Block #2) via shared memory and C++ Library 1 (Block #3) via C# wrapper, and finally, to the dual Novint Falcon robots via USB protocol.

Moreover, the main application is connected to the C++ Library 2 (Block #4) via C# wrapper, and afterward, Block #4 is connected to the Quarc Simulink^©^ via shared memory. Consequently, Quarc Simulink^©^ is connected to the three robots including two Phantoms and one Quanser® via the FireWire® protocol. All five blocks shown in Figure A2 are written by the authors of this article. It is worth mentioning that the quarc block in MATLAB Simulink^©^ has been used for the real-time implementation of this system. For lack of space, the functionality of the software modules will not be discussed in this paper.
